# Supplementary material for: Structure and function of Full-length Tau
Source: PLoS One. 2025 Oct 31;20(10):e0335251. doi: 10.1371/journal.pone.0335251 (PMC12578172; doi:10.1371/journal.pone.0335251)

Structure and function of full-length Tau

Laura Vallés-Saiz [1], Indalo Domene-Serrano[1,2], Ángel J Picher[3], Mar Pérez[4], Vega García-Escudero[1,4,5], Félix Hernández[1]and Jesús Avila[1,5*]

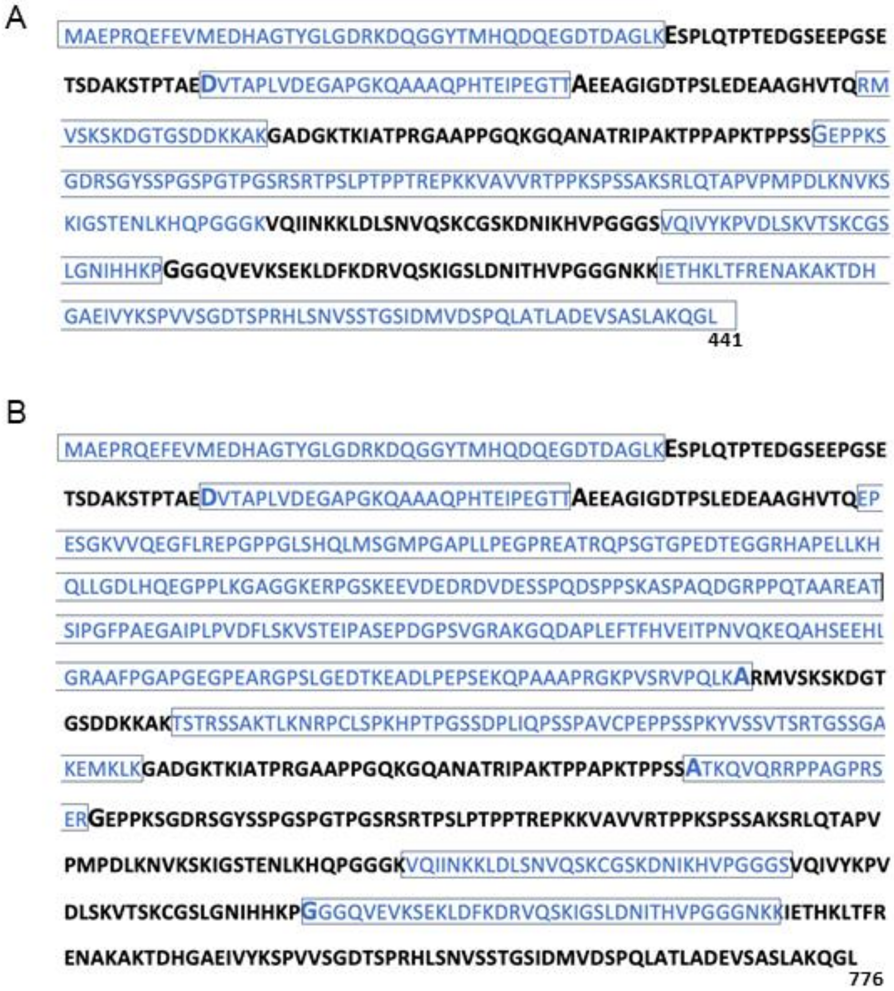

Supplement: S1 File — (A) Amino acid sequence of Tau 4R2N (+2, + 3, + 10). (B) Amino acid sequence of Full Tau (+2, + 3, + 4A, + 6, + 8), an isoform including all exons. Exon 1 starts in blue and alternates with black. Amino acids with a larger font size indicate a codon spanning both exons. Sequences were obtained from ENSEMBL and Uniprotein. (PDF) [file pone.0335251.s001.pdf]
