# Supplementary figures and images for: Structure and function of Full-length Tau
Source: PLoS One. 2025 Oct 31;20(10):e0335251. doi: 10.1371/journal.pone.0335251 (PMC12578172; doi:10.1371/journal.pone.0335251)

A

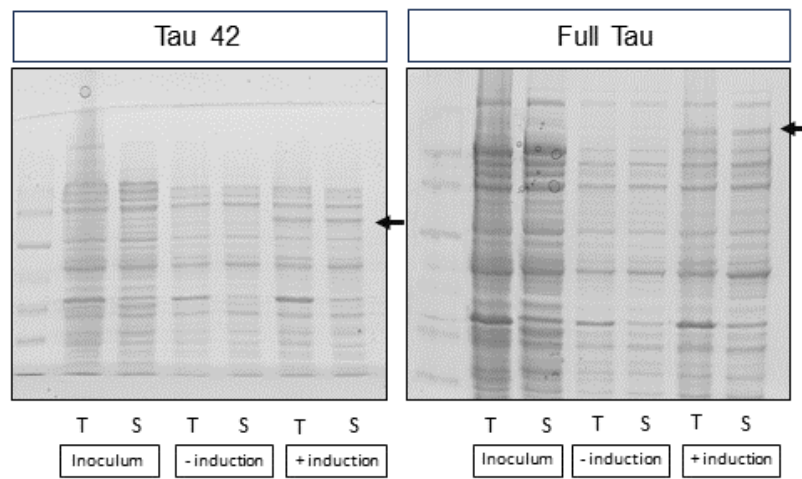

B

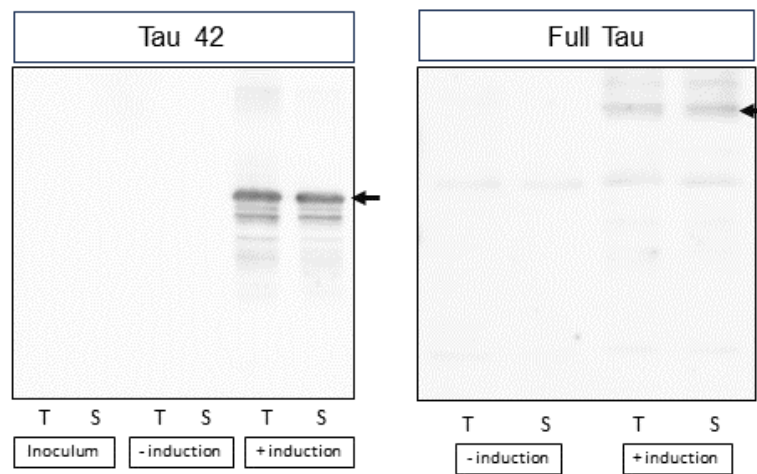

Supplement: S2 File — Characterization by gel electrophoresis of Full human Tau and Tau 4R2N isoforms purified from bacteria (see Methods). (A) Coomassie brilliant blue staining. (B) Western Blot with antibody HT7. (PDF) [file pone.0335251.s002.pdf]

A

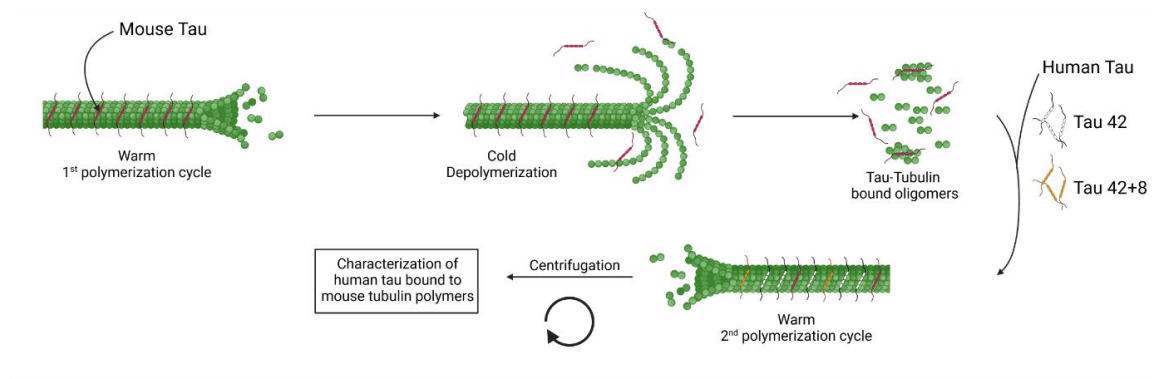

B

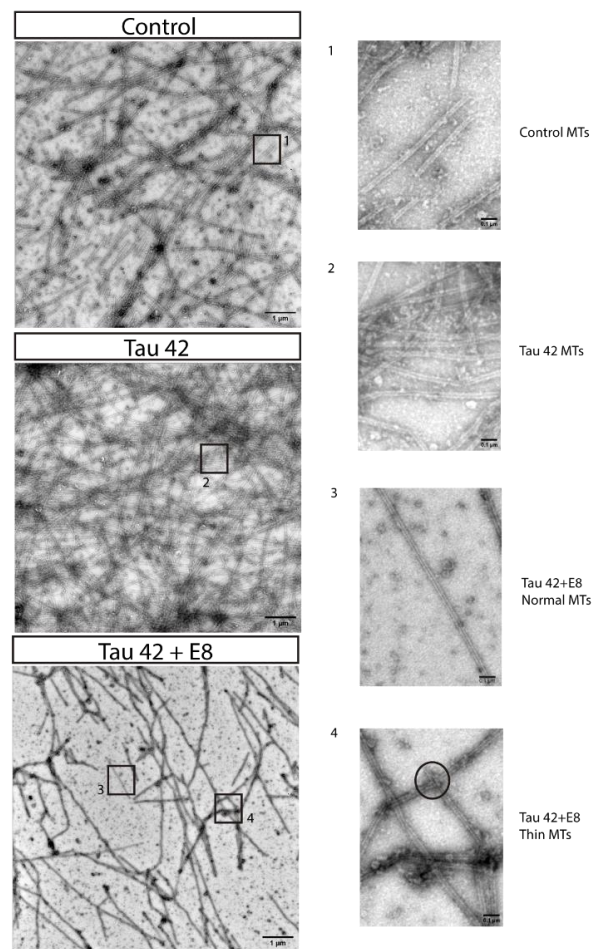

Supplement: S3 File — Electron microscopy images of second polymerization cycle of mouse brain microtubules in the absence (control) or presence of Tau 4R2N or Exon 8 Tau isoform. Circle points thin MTs. (PDF) [file pone.0335251.s003.pdf]

# Full Tau

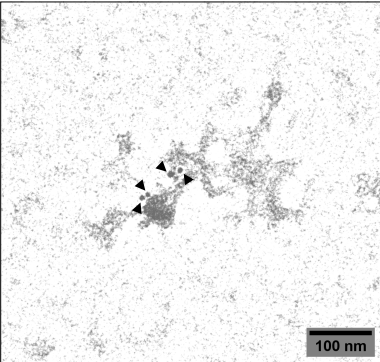

Supplement: S4 File — Immunogold labeling of Full Tau aggregates corresponding to the analysis shown in Figure 3 (white arrows indicate nanogold particles). (PDF) [file pone.0335251.s004.pdf]

Tau 4R2N

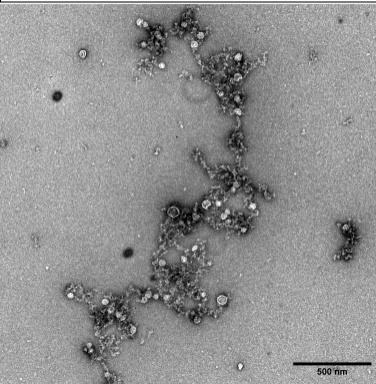

Full Tau

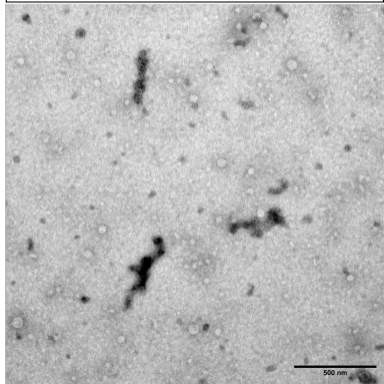

Supplement: S5 File — (A) Aggregation assays of Full Tau and Tau 4R2N carried out without heparin. (PDF) [file pone.0335251.s005.pdf]

Fig.1 B

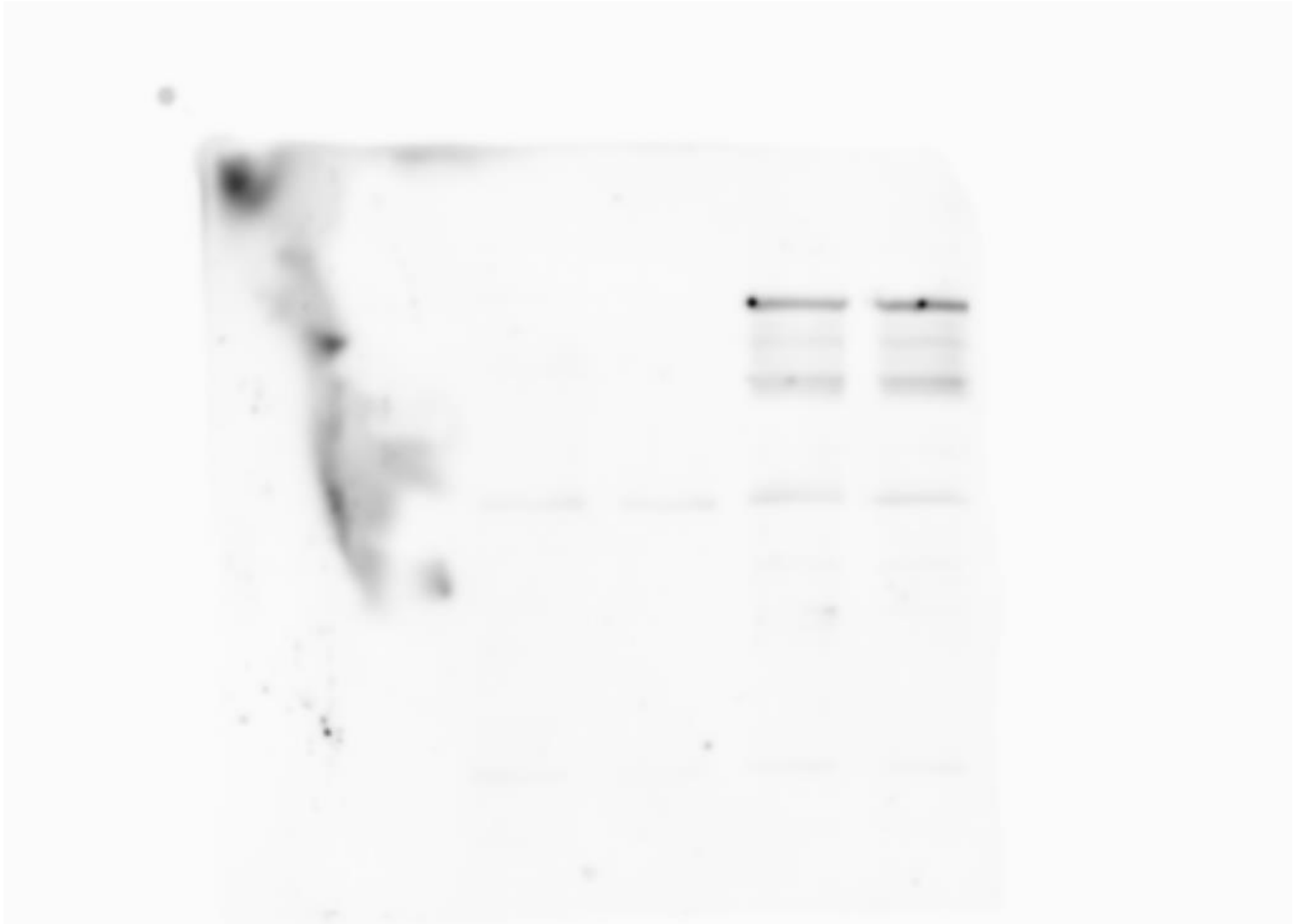

Fig.1 B

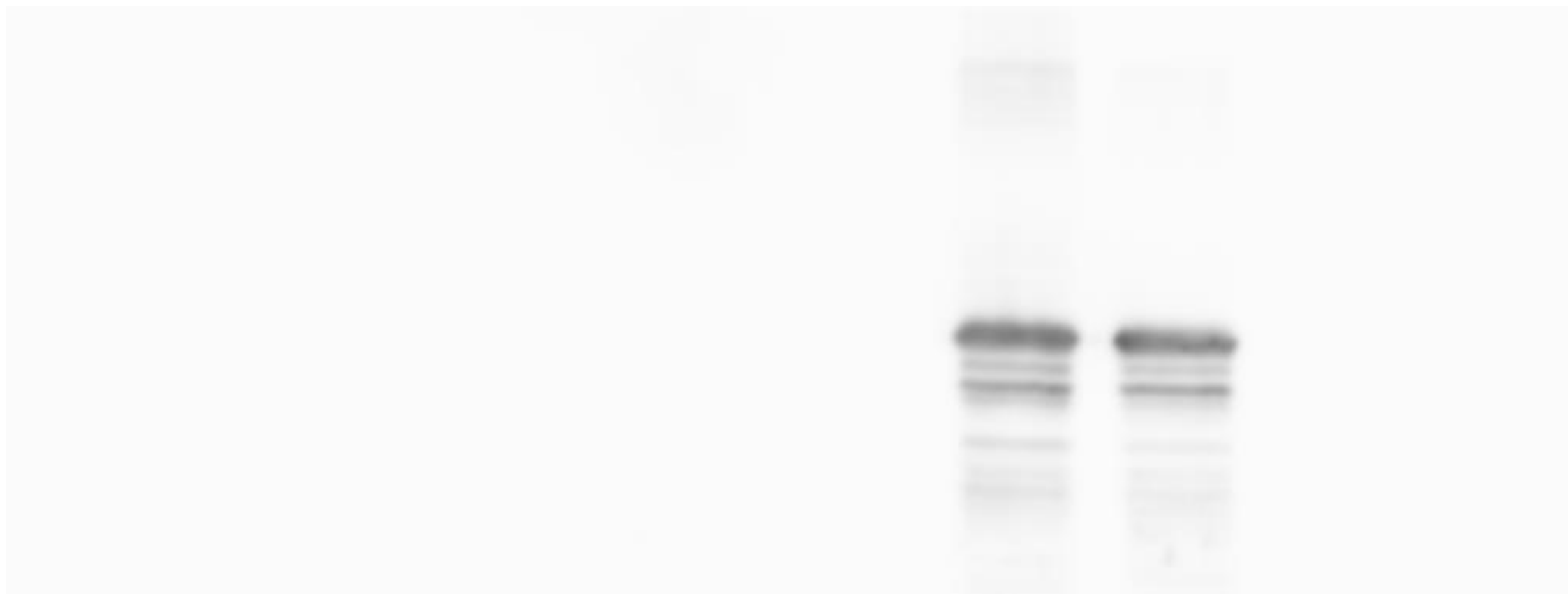

Fig.1 C

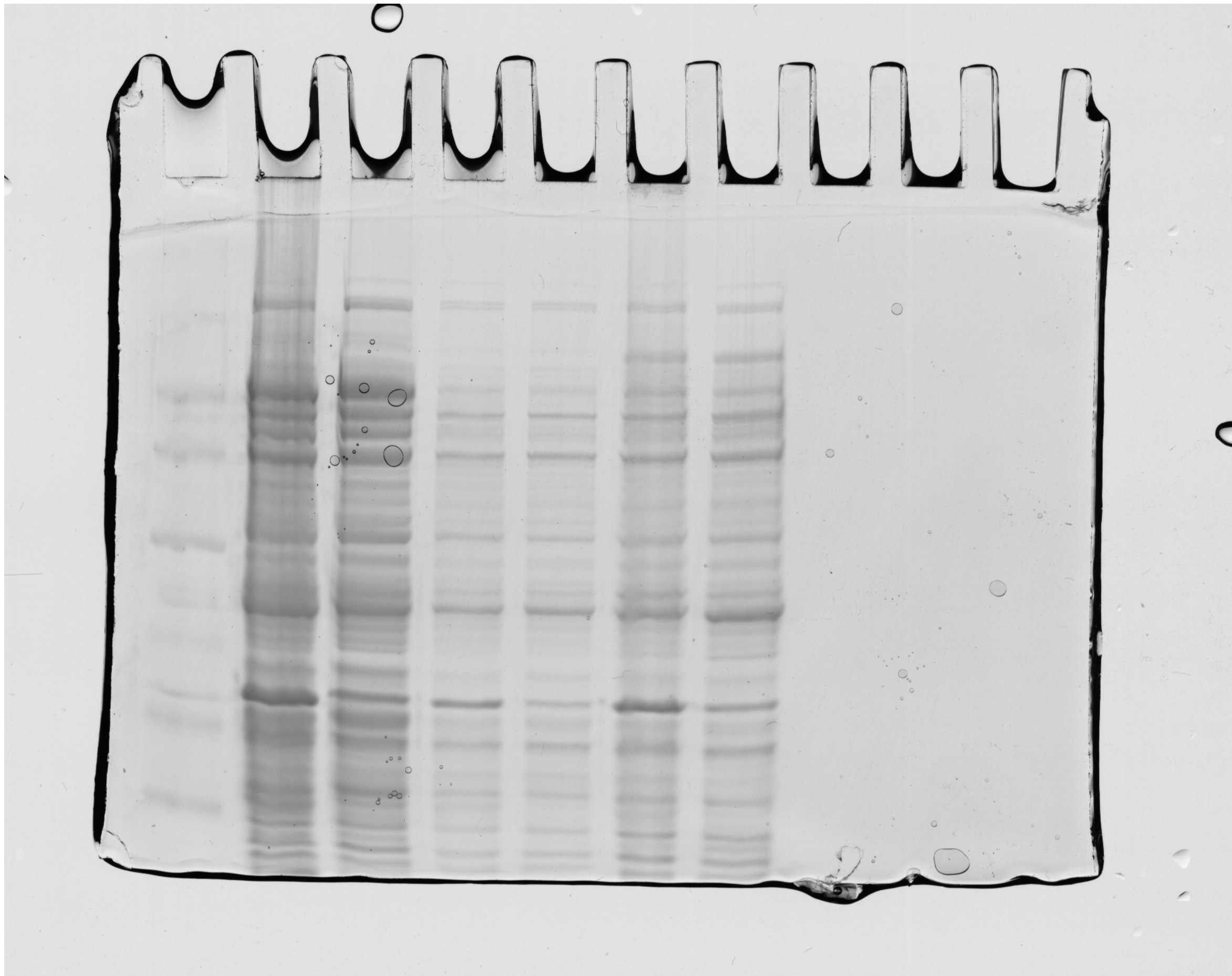

Fig.1 C

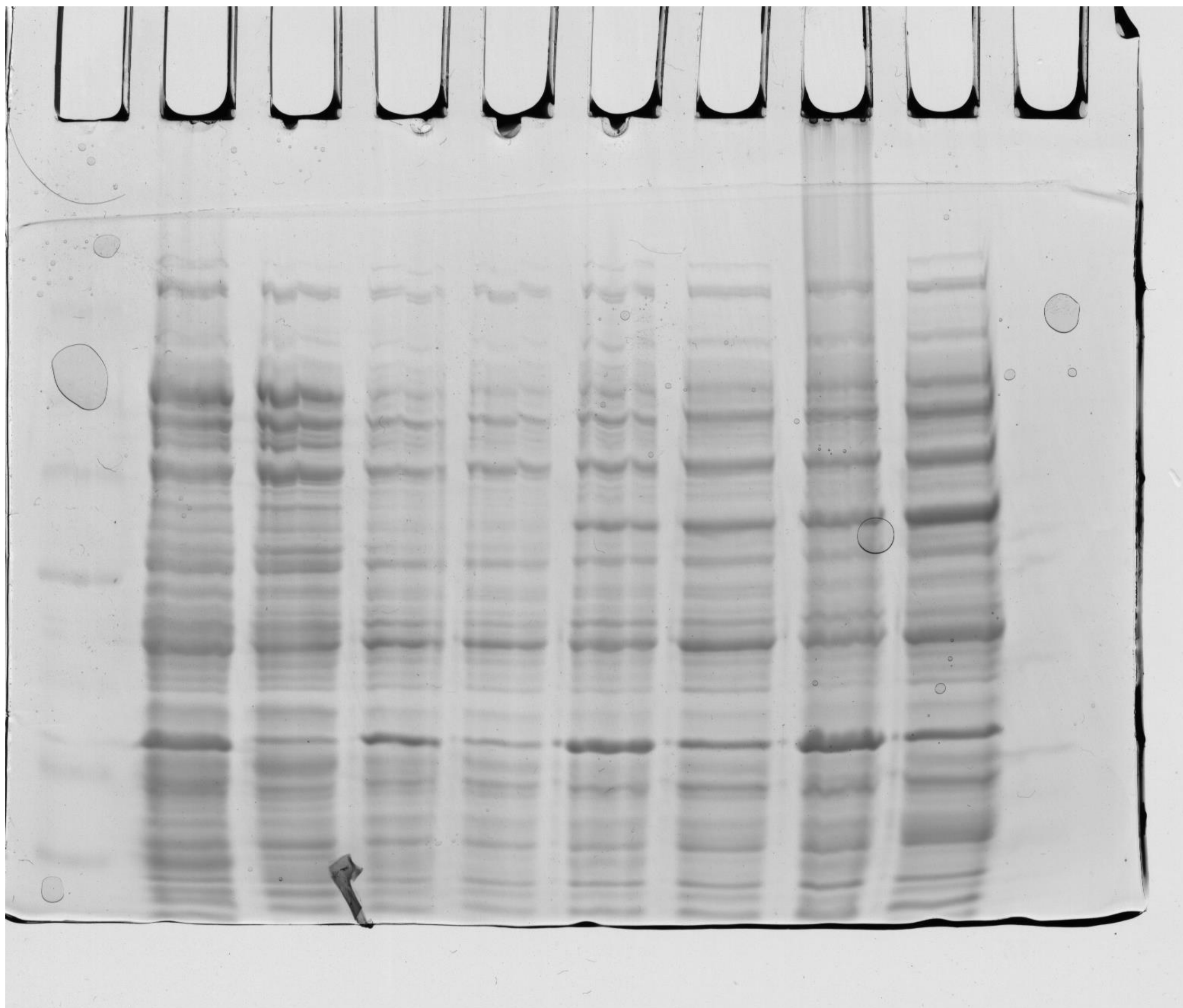

Fig. 2 D

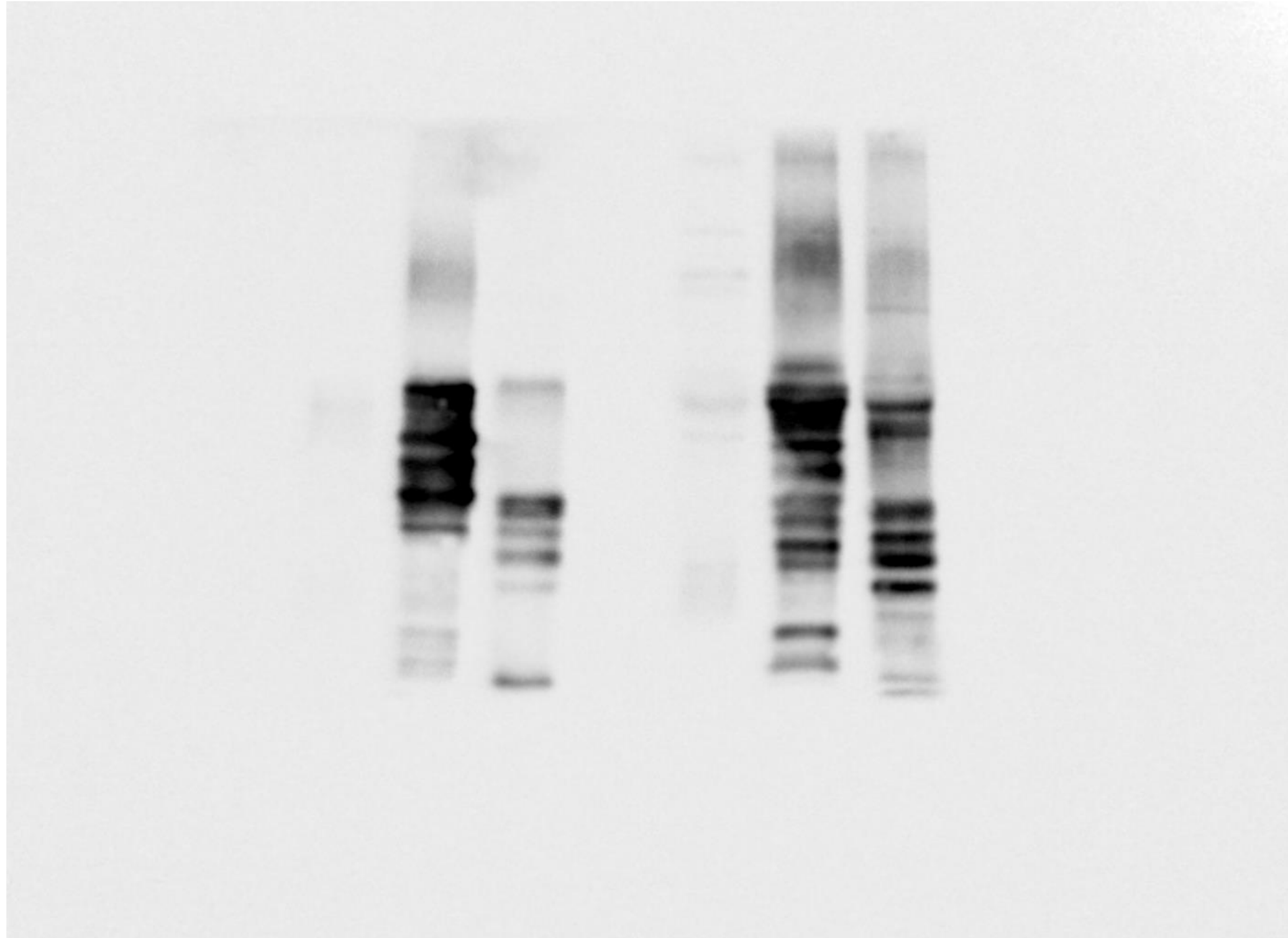

Fig. 2 D

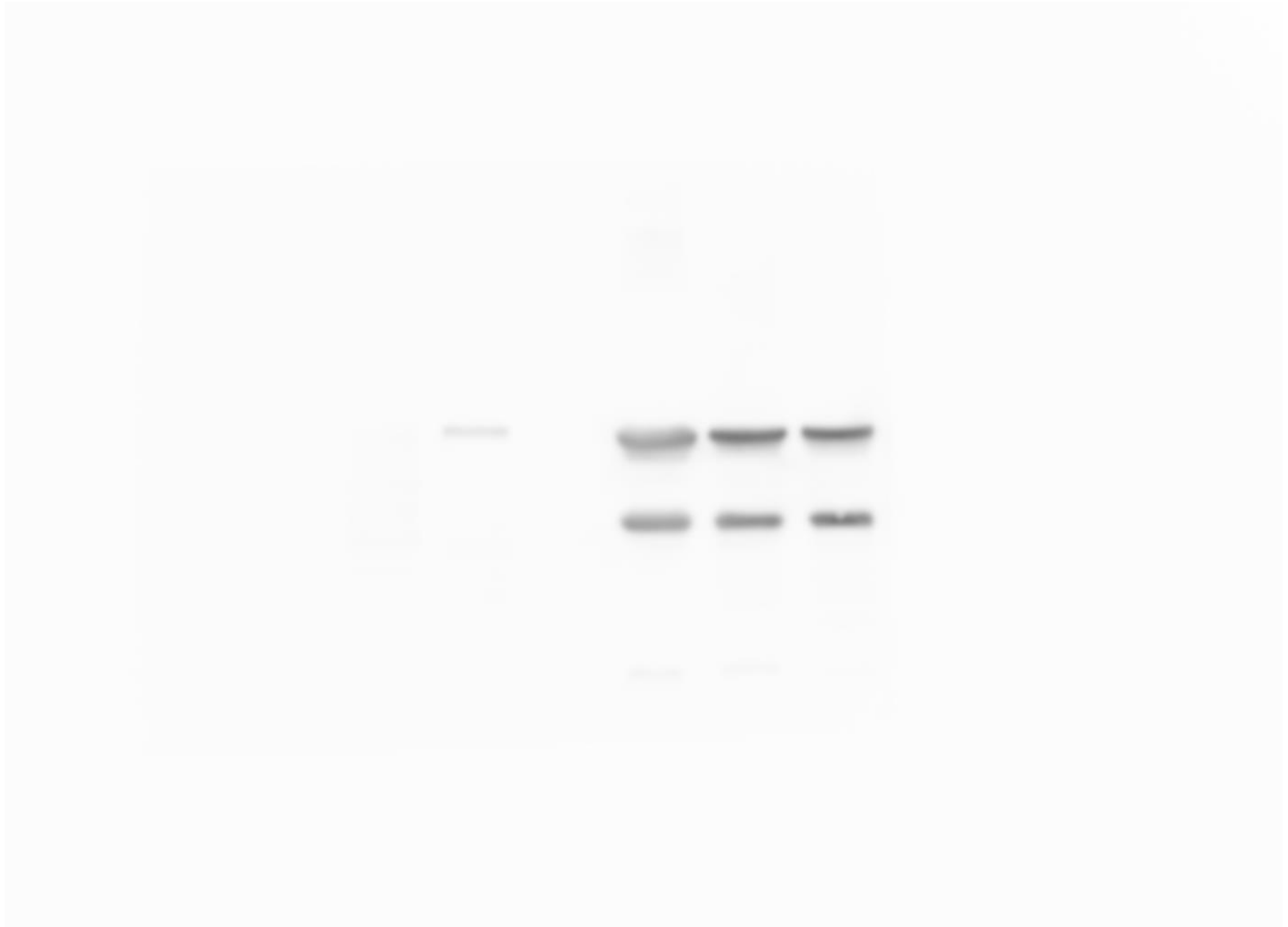

Fig. 3 F

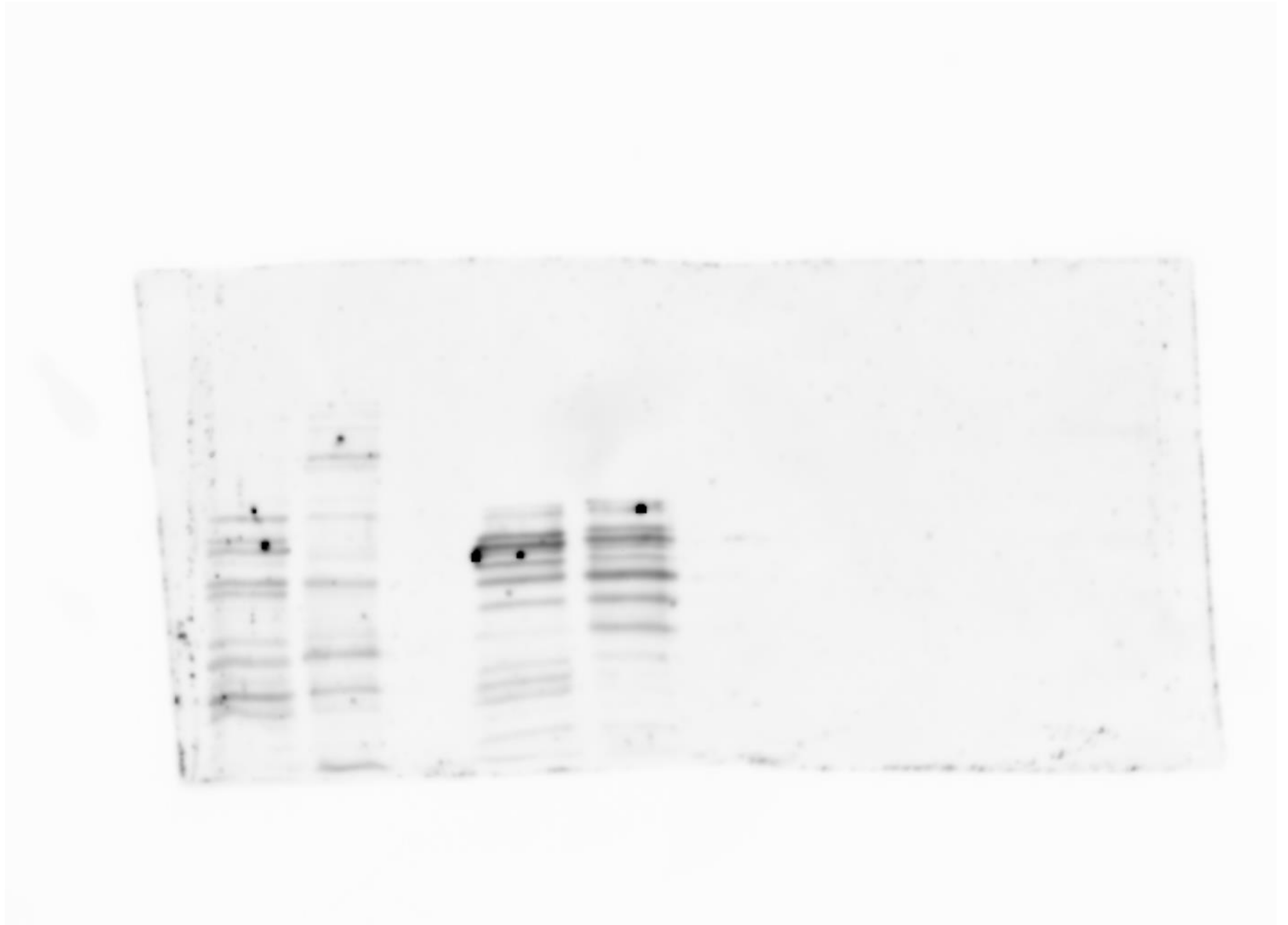

Supplement: S7 File — This figure includes the unprocessed original images from all key experiments, including Western blots and Coomassie-stained gels. (PDF) [file pone.0335251.s007.pdf]
