# Supplementary material for: Structure and function of Full-length Tau
Source: PLoS One. 2025 Oct 31;20(10):e0335251. doi: 10.1371/journal.pone.0335251 (PMC12578172; doi:10.1371/journal.pone.0335251)
Supplement: S6 File — Scheme of the comparison between the sequences of Exon 8 and Exons 9–10 (Exon 9-gray, Exon 10-black) where basic amino acids are highlighted inside a box showing their similar location in both sequences. (PDF) [file pone.0335251.s006.pdf]

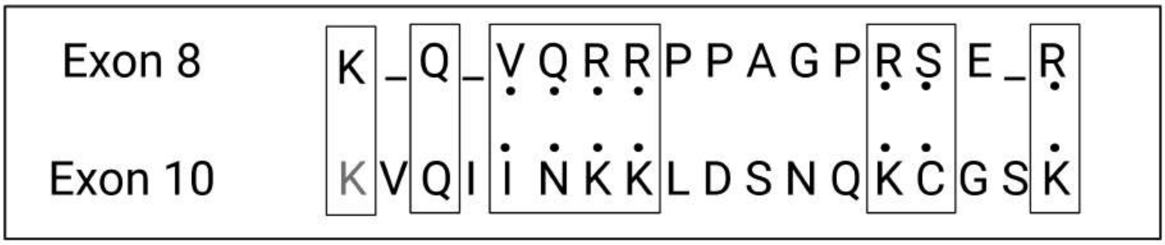
Exon 8
K _ Q _ V Q R R P P A G P R S E _ R
Exon 10
K V Q I I N K K L D S N Q K C G S K
