# Supplementary material for: Structure and function of Full-length Tau
Source: PLoS One. 2025 Oct 31;20(10):e0335251. doi: 10.1371/journal.pone.0335251 (PMC12578172; doi:10.1371/journal.pone.0335251)
Supplement: S1 Table — Comparative of Solvent Accessible Surface Areas of both isoforms. (PDF) [file pone.0335251.s008.pdf]

	Full Tau	Tau 4R2N
**POLAR area/energy**	40355.73	21928.34
**APOLAR area/energy**	59705.34	34076.97
**Total area/energy**	100061.07	56005.32
**Number of surface atoms**	5301	2925
**Number of buried atoms**	384	286
**Buried fraction**	6.8%	8.9%

S1 able. Solvent Accessible Surface Areas Comparative of Solvent Accessible Surface Areas of both isoforms
