# Supplementary material for: Structure and function of Full-length Tau
Source: PLoS One. 2025 Oct 31;20(10):e0335251. doi: 10.1371/journal.pone.0335251 (PMC12578172; doi:10.1371/journal.pone.0335251)
Supplement: Attachment — Submitted filename: PONE-D-25-07400(1).pdf [file pone.0335251.s009.pdf]

PLOS ONE

Structure and function of full-length Tau

--Manuscript Draft--

**Manuscript Number:**	PONE-D-25-07400
**Article Type:**	Research Article
**Full Title:**	Structure and function of full-length Tau
**Short Title:**	Full-length Tau
**Corresponding Author:**	Jesús Avila<br>Severo Ochoa Molecular Biology Centre: Universidad Autonoma de Madrid Centro de Biologia Molecular Severo Ochoa<br>Madrid, SPAIN
**Keywords:**	Tau protein; protein structure; microtubule; protein aggregation; structure-function
**Abstract:**	Tau protein, encoded by the MAPT gene, is a microtubule-associated protein involved in the regulation of microtubule stability in neurons, contributing to cell shape maintenance and intracellular transport, among other functions. Tau is not found as an unique isoform; instead, different tau isoforms of varying sizes are present in the brain but never a full-length tau isoform (Full Tau) containing all its 16 exons has been previously found.<br>This study has explored the structure and function of the full tau isoform, which includes all exons of the MAPT gene. To achieve this, we expressed the full tau isoform in bacteria, alongside the tau 42 isoform as a control and tested its microtubule-binding capacity, self-aggregation propensity, and effects on cultured cells, regarding cell proliferation and cell death. Our results have indicated several differences between the full tau and tau 42 isoforms, suggesting distinct roles in cellular dynamics. To explain those differences, we suggest the role of exon 8, which is present in the full tau isoform but absent in tau 42.
**Order of Authors:**	Laura Vallés-Saiz
	Indalo Domene-Serrano
	Angel J. Picher
	Mar Pérez
	Vega García-Escudero
	Felix Hernández
	Jesús Avila
**Additional Information:**	
**Question**	**Response**
**Financial Disclosure**<br><br>Enter a financial disclosure statement that describes the sources of funding for the work included in this submission. Review the submission guidelines for detailed requirements. View published research articles from *PLOS ONE* for specific examples.<br><br>This statement is required for submission and **will appear in the published article** if the submission is accepted. Please make	Yes

Powered by Editorial Manager® and ProduXion Manager® from Aries Systems Corporation

sure it is accurate.<br><br>**Funded studies**<br>Enter a statement with the following details:<br>• Initials of the authors who received each award<br>• Grant numbers awarded to each author<br>• The full name of each funder<br>• URL of each funder website<br>• Did the sponsors or funders play any role in the study design, data collection and analysis, decision to publish, or preparation of the manuscript?<br><br>Did you receive funding for this work?	
Please add funding details.<br>as follow-up to "**Financial Disclosure**<br><br>Enter a financial disclosure statement that describes the sources of funding for the work included in this submission. Review the submission guidelines for detailed requirements. View published research articles from *PLOS ONE* for specific examples.<br><br>This statement is required for submission and **will appear in the published article** if the submission is accepted. Please make sure it is accurate.<br><br>**Funded studies**<br>Enter a statement with the following details:<br>• Initials of the authors who received each award<br>• Grant numbers awarded to each author<br>• The full name of each funder<br>• URL of each funder website<br>• Did the sponsors or funders play any role in the study design, data collection and analysis, decision to publish, or preparation of the manuscript?<br><br>Did you receive funding for this work?"	This work has been supported by grants from the Spanish Ministry of Science: PID2020-113204GB-I00 (F.H.) and PID2021-123859OB-100 from MCIN /AEI/10.13039/501100011033 / FEDER, UE (J.A.). The Centro de Biología Molecular Severo Ochoa (CBMSO) is a Severo Ochoa Center of Excellence (MICIN, award CEX2021-001154-S).
Please select the country of your main research funder (please select carefully	SPAIN - ES

as in some cases this is used in fee calculation).<br>as follow-up to "**Financial Disclosure**<br><br>Enter a financial disclosure statement that describes the sources of funding for the work included in this submission. Review the submission guidelines for detailed requirements. View published research articles from *PLOS ONE* for specific examples.<br><br>This statement is required for submission and **will appear in the published article** if the submission is accepted. Please make sure it is accurate.<br><br>**Funded studies**<br>Enter a statement with the following details:<br>• Initials of the authors who received each award<br>• Grant numbers awarded to each author<br>• The full name of each funder<br>• URL of each funder website<br>• Did the sponsors or funders play any role in the study design, data collection and analysis, decision to publish, or preparation of the manuscript?<br><br>Did you receive funding for this work?"	
**Competing Interests**<br><br>Use the instructions below to enter a competing interest statement for this submission. On behalf of all authors, disclose any competing interests that could be perceived to bias this work—acknowledging all financial support and any other relevant financial or non-financial competing interests.<br><br>This statement is required for submission and **will appear in the published article** if the submission is accepted. Please make sure it is accurate and that any funding sources listed in your Funding Information later in the submission form are also	The authors have declared that no competing interests exist.

declared in your Financial Disclosure statement.<br><br>View published research articles from *PLOS ONE* for specific examples.<br><br>**NO authors have competing interests**<br><br>Enter: *The authors have declared that no competing interests exist.*<br><br>**Authors with competing interests**<br><br>Enter competing interest details beginning with this statement:<br><br>*I have read the journal's policy and the authors of this manuscript have the following competing interests: [insert competing interests here]*<br><br>* typeset	
**Ethics Statement**<br><br>Enter an ethics statement for this submission. This statement is required if the study involved:<br><br>• Human participants<br>• Human specimens or tissue<br>• Vertebrate animals or cephalopods<br>• Vertebrate embryos or tissues<br>• Field research<br><br>Write "N/A" if the submission does not require an ethics statement.<br><br>General guidance is provided below. Consult the submission guidelines for detailed instructions. **Make sure that all information entered here is included in the Methods section of the manuscript.**	N/A

Powered by Editorial Manager® and ProduXion Manager® from Aries Systems Corporation

**Format for specific study types**<br><br>**Human Subject Research (involving human participants and/or tissue)**<br>• Give the name of the institutional review board or ethics committee that approved the study<br>• Include the approval number and/or a statement indicating approval of this research<br>• Indicate the form of consent obtained (written/oral) or the reason that consent was not obtained (e.g. the data were analyzed anonymously)<br><br>**Animal Research (involving vertebrate animals, embryos or tissues)**<br>• Provide the name of the Institutional Animal Care and Use Committee (IACUC) or other relevant ethics board that reviewed the study protocol, and indicate whether they approved this research or granted a formal waiver of ethical approval<br>• Include an approval number if one was obtained<br>• If the study involved *non-human primates*, add *additional details* about animal welfare and steps taken to ameliorate suffering<br>• If anesthesia, euthanasia, or any kind of animal sacrifice is part of the study, include briefly which substances and/or methods were applied<br><br>**Field Research**<br><br>Include the following details if this study involves the collection of plant, animal, or other materials from a natural setting:<br>• Field permit number<br>• Name of the institution or relevant body that granted permission	
**Data Availability**<br><br>Authors are required to make all data underlying the findings described fully available, without restriction, and from the time of publication. PLOS allows rare exceptions to address legal and ethical concerns. See the PLOS Data Policy and FAQ for detailed information.	Yes - all data are fully available without restriction

A Data Availability Statement describing where the data can be found is required at submission. Your answers to this question constitute the Data Availability Statement and **will be published in the article**, if accepted.<br><br>**Important:** Stating 'data available on request from the author' is not sufficient. If your data are only available upon request, select 'No' for the first question and explain your exceptional situation in the text box.<br><br>Do the authors confirm that all data underlying the findings described in their manuscript are fully available without restriction?	
**Describe where the data may be found in full sentences. If you are copying our sample text, replace any instances of XXX with the appropriate details.**<br><br>• If the data are **held or will be held in a public repository**, include URLs, accession numbers or DOIs. If this information will only be available after acceptance, indicate this by ticking the box below. For example: *All XXX files are available from the XXX database (accession number(s) XXX, XXX.*).<br>• If the data are all contained **within the manuscript and/or Supporting Information files**, enter the following: *All relevant data are within the manuscript and its Supporting Information files.*<br>• If neither of these applies but you are able to provide **details of access elsewhere**, with or without limitations, please do so. For example:<br><br>*Data cannot be shared publicly because of [XXX]. Data are available from the XXX Institutional Data Access / Ethics Committee (contact via XXX) for researchers who meet the criteria for access to confidential data.*<br><br>*The data underlying the results presented in the study are available from (include the name of the third party*	Data is available in data repository: https://doi.org/10.6084/m9.figshare.28351508.v1

and contact information or URL).<br>• This text is appropriate if the data are owned by a third party and authors do not have permission to share the data.<br><br>* typeset	
Additional data availability information:	Tick here if the URLs/accession numbers/DOIs will be available only after acceptance of the manuscript for publication so that we can ensure their inclusion before publication.

Powered by Editorial Manager® and ProduXion Manager® from Aries Systems Corporation

Manuscript

Click here to access/download;Manuscript;Valles-Saiz_Manuscript.docx

Structure and function of full-length Tau

Laura Vallés-Saiz [1], Indalo Domene-Serrano[1,2], Angel J. Picher[3], Mar Pérez[4], Vega García-Escudero[1,4,5], Félix Hernández[1] and Jesús Avila[1,5*]

[1] Centro de Biología Molecular Severo Ochoa, CSIC-UAM), 28049 Madrid, Spain.

[2] Facultad de Ciencias Experimentales, Universidad Francisco de Vitoria, Pozuelo de Alarcon, 28223 Madrid, Spain.

[3] Expedeon S.L.U., Parque Científico de Madrid, Cantoblanco, Madrid, Spain.

[4] Anatomy, Histology and Neuroscience Department, School of Medicine, Universidad Autónoma de Madrid (UAM), 28029, Madrid, Spain

[5] Networking Research Centre on Neurodegenerative Diseases (CIBERNED), Instituto de Salud Carlos III, 28029 Madrid, Spain

*Correspondence: Jesús Ávila, Centro de Biología Molecular Severo Ochoa, CSIC-UAM, C/Nicolás Cabrera 1, 28049 Madrid, Spain. Email: javila@cbm.csic.es

Running title: Full-length Tau

Abstract

Tau protein, encoded by the MAPT gene, is a microtubule-associated protein involved in the regulation of microtubule stability in neurons, contributing to cell shape maintenance and intracellular transport, among other functions. Tau is not found as an unique isoform; instead, different tau isoforms of varying sizes are present in the brain but never a full-length tau isoform (Full Tau) containing all its 16 exons has been previously found.

This study has explored the structure and function of the full tau isoform, which includes all exons of the MAPT gene. To achieve this, we expressed the full tau isoform in bacteria, alongside the tau 42 isoform as a control and tested its microtubule-binding capacity, self-aggregation propensity, and effects on cultured cells, regarding cell proliferation and cell death. Our results have indicated several differences between the full tau and tau 42 isoforms, suggesting distinct roles in cellular dynamics. To explain those differences, we suggest the role of exon 8, which is present in the full tau isoform but absent in tau 42.

Keywords: Tau protein; protein structure; microtubule; protein aggregation; structure-function.

Introduction

Tau protein is one of the brain microtubule-associated proteins present in neuronal cells [1, 2]. Tau is crucial for regulating microtubules stabilization [3], tubular structures that are part of the cytoskeleton and are essential for maintaining cell shape and for intracellular transport. In the brain, tau regulates microtubule stabilization that are critical for the development and maintenance of neuronal connections [4-9]. The gene that encodes the tau protein is MAPT gene, located on chromosome 17q21 [10]. Three main Tau transcripts from the MAPT gene have been described [11]. These RNA transcripts translate into different Tau protein isoforms, but none have been found to translate the full-length Tau protein [11].

In the human central nervous system (CNS), six main distinct tau isoforms are expressed, each contributing to functional diversity and complexity in neuronal operations [4, 12, 13]. These isoforms share a set of constitutive exons—1, 4, 5, 7, 9, 11, 12, and 13—that are consistently present across all variants, demonstrating their essential roles in tau protein structure. Conversely, the non-constitutive exons 2, 3, and 10, are included in specific isoforms, reflecting their regulatory roles in isoform-specific functions [4, 5, 12]. These isoforms arise through alternative splicing. Where there are inclusion or exclusion of specific exons by proteins that either promote or suppress inclusion [14]. The generation of diverse protein isoforms from a single gene via alternative splicing is not as common as one might believe. RNA-seq studies indicate that almost all coding genes have a primary protein isoform, and only a few genes show strong evidence for multiple alternative splice isoforms, with MAPT being one of the well-studied examples [15].

Furthermore, recent studies have described new tau isoforms obtained through intron retention rather than alternative splicing. These isoforms retain intron 3, 11, or 12 [16-

18]. This observation suggests that exons 1, 12, and 13 may be considered non-constitutive exons. Despite the discovery of these new isoforms, none results in a transcript containing all 16 exons of the MAPT gene.

There are two types of Nervous System. First, CNS develops after the formation of the neural tube. Afterwards, cells from neuronal crest migrate to form the Peripheral Nervous System (PNS) [19]. Sometimes CNS and PNS are considered as different entities with transitions between both systems [20], such as in the case of dorsal root ganglion [21]. Additionally, specific tau isoforms are present in mammal peripherical nervous system, where exons 4A and 6 are found as Big Tau isoform [22, 23]. This big tau isoform, may be protective for tau misfolding, less vulnerable to tau pathology and, as indicated, binds to microtubules from PNS [6]. The presence of exon 8 has been detected in cDNA derived from bovine RNA transcripts [24, 25]. Also, exon trapping assays indicate that the exclusion of this exon is the default splicing pattern [5, 26]. However, the inclusion of exon 8 in mature RNA transcripts has been observed not only in bovine but also in the monkey brain [27]. This pattern contrasts significantly with findings in humans, where exon 8 is typically not included in mature tau isoforms, highlighting a notable difference in RNA processing between species [24].

Although the ENSEMBL database (MAPT-205; ENSP00000410838.2) indicates that a transcript containing all the human Tau exons capable of being translated into proteins can exist, there are currently no reports of its expression. In this work, we will examine the properties, structure and functions of Full Tau RNA transcript contains 16 exons: -1, 1, 2, 3, 4, 4A, 5, 6, 7, 8, 9, 10, 11, 12, 13 and 14, totaling 776 amino acids (known in this work as Full Tau; **Fig. 1B**). Exon -1 is part of the promoter and is transcribed but not translated, similar to exon 14 [28].

Figure 1: Tau 42 and Full Tau protein purification. (A) Alternative splicing scheme of Tau 42 and Full Tau and specific domains. **(B)** Coomassie brilliant blue staining of purification of both proteins showing control of both expressions without and with

induction of expression using 0.5mM IPTG. **(C)** Western Blot using the antibody that recognizes human tau (HT7) indicating bands from induction are corresponding tau.

Results

Isolation and purification of Full Tau protein isoform

As previously indicated, we have focused this work on the tau protein isoform containing all the exons. We searched databases such as Uniprot, NCBI, and ENSEMBL to look for the presence of Full Tau. Thus, Full Tau protein could be found in ENSEMBL under the identifier (MAPT-205; ENSP00000410838.2) but it seems that could be present in humans and other mammals, in a very low amount, not only at protein but also at RNA level. Obviously, the translated protein should be very low when the expression of its RNA is also very low. Thus, to isolate full tau protein, we have synthetized the cDNA for the RNA expressing all the exons and we include that cDNA in a plasmid to be expressed in bacteria. As control we did the same experiment to express human Tau 42 isoform [29].

Figure 1 shows Tau 42 molecule and Full Tau molecule (**Fig. 1A**), these proteins were compared to identify structural differences. The complete amino acid sequences of both human Tau isoforms are shown in supporting figure 1. Tau 42 includes exons +2, +3, and +10 (**Supp. Fig. 1A**) while Full Tau additionally incorporates exons +4A, +6, and +8, with a total of 776 aminoacids (**Supp. Fig. 1B**). In both sequences, the exons are highlighted alternating between blue and black to facilitate identification.

As indicated, we induced the expression of both proteins in *E. coli* BL21 strains for subsequent *in vitro* analyses. The isolation of isoforms is well described in Material &

Methos. **Figure 1B** shows the Coomassie-stained gel control of both expressions without and with induction of expression using 0.5mM IPTG. In section C, the Western Blot using the antibody that recognizes human tau (HT7) confirms that the new bands are tau. Both Coomassie-stained gel control and Western Blot raw images are in **Supplementary Figure 2 A-B**. The results highlight the efficiency of the inducible expression system used, ensuring that overexpression of the isoforms of interest occurs only under controlled conditions. The clear differentiation in the amino acid sequences between Tau42 and Full Tau underscores the importance of studying these isoforms individually to understand their specific roles.

Full Tau, microtubule binding capacity

The primary and most well-known function of the Tau protein is its binding to microtubules [4]. To confirm the *in silico* findings, we decided to study the microtubule-binding capacity of our Full Tau isoform by separating microtubule-bound and free Tau by *in vitro* polymerization, followed by electron microscopy analysis and by separating polymerized or aggregated from soluble protein through centrifugation [30].

To analyze the binding of full human Tau to microtubules and compare it with the binding of the human Tau 42 isoform, we used the same microtubules. These microtubules are mouse brain microtubules (MTs) obtained after a first polymerization cycle (see scheme in **Fig. 2A**). These mouse brain MTs already contained mouse Tau protein. After cold depolymerization, tubulin-Tau links remain during a second polymerization cycle [2]. Before this second cycle, equal amounts of Full Tau or Tau 42 isoforms were added to depolymerized mouse microtubules. The second cycle was then carried out, allowing the added human Tau isoforms to bind to the second-cycle polymerized MT at the open sites present in the pre-assembled MT [31].

Figure 2: Microtubule binding capacity of Full Tau isoform: (A) Scheme of the interaction of human Tau isoforms to second polymerization cycle of mouse microtubules (see Methods). **(B)** Electron microscopy images of second polymerization cycle of mouse brain microtubules in the absence (control) or presence of Tau 42 or Full Tau isoform. Circle points thin MTs. **(C)** Coomassie brilliant blue staining showing tubulin percentage after centrifugation of the two cycle-polymerization MTs. **(D)** Western Blot analyses of human Tau bound to microtubules after copolymerization, in the second polymerization cycle of mouse microtubules, detected by anti-human Tau antibody HT7. **(E)** Quantification of the ratio between Tau 42 and Full Tau Western Blot signal intensity in C. Quantitative analysis shows the mean ± SEM. * $p < 0.05$; ** $p < 0.01$ using one-way ANOVA; post-hoc Turkeys's test, followed by Student's t-test for comparisons. Sample size: n= 3.

After polymerization, the assembled MTs were visualized by electron microscopy (**Fig. 2B**). In MTs in the absence of human tau isoforms or in the presence of human Tau 42, MTs are clearly formed. However, in the presence of human Full Tau some differences in the amount and structure of MT were observed. A lower amount of MTs was found compared with those found in the presence of Tau 42 and a higher proportion of amorphous aggregates were observed (**Fig. 2B**). Microtubule-like polymers with a shorter diameter that control microtubules were also found (**Fig. 2B**). All of these observations indicate that the presence of Full Tau isoform may partially interfere with the previously reported *in vitro* MTs polymerization taking place in the absence of that isoform.

Tubulin polymers in the absence or presence of human tau isoforms, could be isolated by centrifugation and in the pelleted fraction, the presence of tubulin can be visualized by fractionation of the MTs proteins by gel electrophoresis, and staining with Coomassie blue (**Fig. 2C**). By comparing the results of Fig 2B and 2C we can conclude that there is

not a main difference in the amount of pelleted protein, but there is a difference at the structural level of those pelleted tubulin aggregates.

How does the presence of exon 8 affect Tau binding to microtubules?

Figures 2D and **2E** demonstrate that Full Tau exhibits reduced binding to microtubules compared to the Tau 42 isoform. Full Tau closely resembles Big Tau, an isoform containing all Tau exons except exon 8 [6]. However, previous studies [32] have shown that Big Tau binds to microtubules with higher affinity to that now found for Full Tau. To investigate the impact of exon 8 on Tau-microtubule binding, we examined the binding of an artificial isoform composed of Tau 42 plus exon 8. The results presented in **Supplementary Figure 3** reveal that this isoform has a significantly reduced microtubule-binding capacity, similar to that found for full tau isoform and different from that of big tau isoforms (32). These findings suggest that the presence of exon 8 decreases the interaction of Tau with microtubules, explaining the weaker microtubule binding observed for Full Tau compared to the Tau 42 isoform.

***In vitro* aggregation of Full Tau**

We decided to study the aggregation capacity of the Full Tau isoform, as this is a crucial aspect in the research of neurodegenerative diseases such as Alzheimer's disease (AD) and other tauopathies [4, 33]. Tau is a microtubule-associated protein that, under pathological conditions, can form insoluble aggregates, a central pathological feature in these tauopathies.

Figure 3A shows representative electron microscopy images illustrating the influence of heparin on the aggregation of Tau 42 and Full Tau isoforms. Due to it is established that the presence of heparin accelerates filament polymerization for tau [34, 35]. In the

presence of heparin, Tau 42 isoform, forms dense and highly organized structures (filaments). On the other hand, Full Tau in the presence of heparin, shows less aggregated structures, that are less organized and more amorphous compared to those assembled in the presence of Tau 42.

Figure 3. Aggregation capacity of T42 and Full Tau isoforms: (A) Representative images of electron microscopy of tau aggregates obtained upon *in vitro* incubation of purified T42 and Full Tau extracts from bacteria in presence of heparin to promote aggregate formation. Filaments marked with a white arrow. Scale bars show 400nm. **(B)** Western Blot of Tau isoforms present in soluble or pellet fractions after centrifugation. **(C)** Quantitative ratio of Tau 42 and Full Tau protein. Quantitative analysis shows the mean ± SEM. * $p < 0.05$; ** $p < 0.01$ using unpaired; 2-tailed t test. Sample size: n= 3.

To quantify the amount of Tau aggregates, following heparin addition and incubation, the samples were centrifuged, and the presence of human Tau isoforms in the supernatant and pellet was determined. **Figure 3B** shows a larger amount of pelleted protein for the Tau 42 isoform compared to the human Full Tau isoform, quantified in **Figure 3C**.

In silico 3D structural analysis of human Tau protein isoforms: Tau 42 and Full Tau

To try to explain the previous, we decided to perform a three-dimensional *in silico* analysis of both isoforms using the models with the highest confidence (highest pLDDT) among the five predictions generated by AlphaFold2 (AF2) [36]. Figure 4 shows the three-dimensional structure predictions of the Tau 42 and Full Tau isoforms, generated with AF2. The microtubule-binding domains (exons 9, 10, 11, and 12) are highlighted in

red, exon 8 is highlighted in green, while the rest of the structure is shown in white. These predictions were visualized at different rotation angles (0°, 90°, 180°, and 270°) around the "y" axis to provide a comprehensive view of the conformation of both isoforms. The images reveal the spatial arrangement of the microtubule-binding domains, which are crucial for the function of Tau in microtubule stabilization.

The analysis reveals that the microtubule-binding domains in Tau 42 are more accessible compared to Full Tau. In all rotations (0°, 90°, 180°, and 270°), the red-highlighted exons of Tau 42 are more prominently exposed and appear less obstructed by other regions of the protein. This suggests that in Tau 42, these domains can more easily interact with microtubules, facilitating a greater binding and stabilization capacity. However, in the Full Tau isoform, these domains seem to be more enveloped or partially covered, mainly exon 8 (**Figure 4,** green). This reduced accessibility could affect its ability to efficiently interact with microtubules.

Figure 4: Tridimensional structure prediction of Tau 42 and Full Tau. Microtubule Binding Domain Exons' 9, 10, 11, 12 are represented in red, the rest of exons in white. Both are the predictions with the highest confidence (highest pLDDT) among the five predictions made by AF2. Images were taken at different degrees of rotation about the "y" axis for complete visualization. Exon 8 is highlighted in green and green arrows. MTBD is colorized in red with red arrow heads. Scheme of proteins structure highlighting differences in both structure and size.

Differential Effects of Tau Isoforms on Cell Viability and Growth Rate in HEK 293T Cells

Neurons exhibit a significantly lower proliferation capacity compared to non-neuronal cells. One possible explanation is that cell proliferation requires dynamic microtubules to facilitate the formation of the mitotic spindle. However, in neurons, microtubules are highly stable and lack such dynamism; interphase microtubules are not depolymerized for subsequent assembly into the mitotic spindle, due to the presence of microtubule-associated proteins (MAPs), such as Tau [37]. Additionally, the stability of neuronal microtubules supports the development of their unique asymmetric morphology, as opposed to the more circular-like morphology typically seen in non-neuronal cells (see reference [4]). Interestingly, when Tau is expressed in non-neuronal cells, some neuronal-like features, can be observed [38]. To investigate this further, we expressed Full Tau and the Tau 42 isoform in a non-neuronal cell line to assess potential changes in cell proliferation.

The representative images in **Figure 5A** show the cellular proliferation of HEK 293T cells after infection with lentiviruses expressing Tau 42 and Full Tau, and its growth rate, quantitatively represented as the percentage of newly occupied area by the cells **(Fig. 5B).** We decided to study cellular proliferation rate as it allows us to identify whether the overexpression of one of the isoforms could generate stress or lead to apoptosis due to excessive microtubule stabilization, which affects their ability to properly adhere, mitotic capacity and maintain their normal extended morphology. It has been previously described that this occurs with the overexpression Tau [38].

Figure 5. Proliferation and viability of HEK 293T cells following lentiviral infection with Tau 42 and Full Tau. **(A)** Cellular proliferation rate, assessed through images taken 24 hours post-transduction and after 48 hours of growth. Scale bar: 40 µm. **(B)** Quantification of cellular proliferation based on the newly occupied area by cells. Sample size: n = 3 fields per replicate (n = 3). **(C)** Calcein viability assay comparing cell death between the negative

control and transduction controls for each protein. Scale bar: 100 µm **(D)** Quantitative analysis of viability, measure from the data shown in (C). Sample size: n = 3 fields per replicate (n = 3). **(E)** Representative images of the calcein viability assay in cells overexpressing Tau 42 and Full Tau, along with their respective transduction controls (C. Empty-Tau 42 and C. Empty-Full Tau). Same scale as (E). **(F)** Quantification of cellular viability the data shown in (E). Sample size: n = 3 fields per replicate (n = 3). Quantitative data are presented as mean ± SEM. Statistical significance: *p < 0.05; **p < 0.01, determined by one-way ANOVA followed by Tukey's post-hoc test, with additional comparisons conducted using Student's t-test. Sample size: n = 5 fields per replicate (n = 3).

In the negative control condition, cells maintain a common growth rate for the cell line. However, cells upon lentiviral infection show proliferative changes. When we quantified these growth rate changes (**Fig. 5B**), we found significant differences when comparing Tau 42 with negative control and Full Tau. However, no significant differences were found between negative control and Full Tau. This could indicate that the Full Tau isoform does not induce microtubule over-stabilization, as occurs with the Tau 42 isoform.

Cell viability was also analyzed using calcein **(Fig. 5C-F)**. Calcein is a hydrophobic, non-fluorescent compound that easily penetrates living cells. Once inside, intracellular esterases hydrolyze calcein, converting it into a hydrophilic, highly fluorescent molecule that remains well-retained within the cytoplasm. First, negative control was compared with empty lentiviral transduction controls, and it was observed that infection caused a significant cell death **(Fig. 5C-D)**. Once the effect of cells transduction was determined, the cell viability of cells overexpressing Tau 42 and Full Tau isoforms was compared. The overexpression of Full Tau isoform did not lead to a reduction in cell viability compared to its infection control (C.Empty-Full Tau) **(Fig. 5E)**. In contrast, the

overexpression of the Tau 42 isoform resulted in a statistically significant increase in cell death compared to its infection control (C.Empty-Tau 42), the negative control, and Full Tau **(Fig. 5E-F)**. These results suggest that the expression of Tau 42 has a greater impact on altering cellular physiology than Full Tau. This is likely due to excessive microtubule stabilization by Tau 42, which interferes with the normal dynamics necessary for cell division and cellular transport. Consequently, we observe distinct differences in how these isoforms affect cellular structure viability and proliferation. These findings highlight the specific role of Tau 42 in disrupting cellular morphology, suggesting its potential impact on cellular functions related to microtubule dynamics.

Discussion

Isolation of microtubules from mammalian brains results in the presence of not only the main component of microtubules, tubulin, but also microtubule-associated proteins (MAPs) such as MAP1A, MAP1B, MAP2, Tau, and others [1, 39, 40]. In all cases, except for the Tau protein, the full-length protein is expressed, despite the presence of shorter isoforms [1, 39-41]. However, there are currently no reports indicating the presence of the full-length Tau isoform in the mammalian brain.

Curiously, shorter tau isoforms are expressed in embryonic or first developmental stages, and larger isoforms are expressed at later times of development and maturation, but even in old stages the whole protein has not been found, probably because the level of its RNA is very low, even at mature stages.

We decided to study a Tau isoform with all exons present in the MAPT gene, identified in ENSEMBL database as MAPT-205; ENSP00000410838.2, which we refer to as Full Tau in this work, and compare it with the well-described Tau 42 isoform [29]. A characteristic of the Full Tau isoform is that it includes all exons, which means the

presence of exon 4A and exon 8 simultaneously. However, it has been described that both exons do not appear together in any known Tau isoform [6, 12], highlighting the potential uniqueness of the Full Tau isoform.

We induced the expression of both isoforms in *E. coli* BL21 strains and conducted various *in vitro* assays with the proteins generated in our laboratory. Given that the microtubule-binding region [42, 43] of tau is crucial for its primary function and aggregation, and our *in-silico* analysis showed it to be less exposed in Full Tau, we performed experiments to evaluate the microtubule-binding capacity of our Full Tau isoform. Electron microscopy showed that, in the absence of added human Tau, the microtubules were well-formed, but their structure was denser and more intertwined in the presence of Tau 42, suggesting a slight promotion of polymerization. Full Tau also slightly promoted polymerization.

Therefore, some differences on the interaction of Full Tau with tubulin to promote MT polymerization can be suggested. Firstly, the inclusion of these additional exons might cause Full Tau to adopt a conformation that partially blocks the microtubule-binding domains, particularly exon 8, as previously suggested. Although these findings are intriguing, more in-depth research is needed to fully understand their implications. Exon 8 sequence has some similarities with Exon 10 sequence (**Supp. Fig. 4**).

In both sequences, basic amino acids could react with the acidic residues present at the C-terminal region of tubulin molecules [44, 45], but with different results: microtubule polymerization or aberrant aggregation (see **Fig. 2B**), could take place in the absence or presence of Full Tau. In addition, Exon 8 could compete with some microtubules binding regions, like exon 10, in its interaction with tubulin monomers. Another possible explanation is that the more complex structure of Full Tau might favor intramolecular interactions that compete with microtubule binding. This means that some regions of the

protein might interact with each other, preventing the microtubule-binding domains from being fully available for their stabilizing function [42, 46, 47]. More work should be done to determine the differences between Full Tau and other Tau isoforms with tubulin.

To study the aggregation capacity, we used heparin, which accelerates the polymerization of Tau filaments [7, 48]. Electron microscopy showed that, in the presence of heparin, Tau 42 formed dense and organized structures, whereas Full Tau formed less organized and more amorphous aggregates, with no filaments found for Full Tau. Also, a lower amount of pelleted aggregates was found in Full Tau compared to Tau 42 isoform. This may be because self-aggregation occurs through the microtubule-binding regions of the Tau molecule, which could be partially obscured by the presence of exon 8, as previously indicated.

In this work, we conducted an *in silico* three-dimensional analysis with AF2 [36], revealing significant structural differences between the two isoforms, particularly in the accessibility or exposure of the microtubule-binding domains. In Tau 42, these domains are more accessible compared to Full Tau, suggesting that Tau 42 may interact more effectively with microtubules. This could be due to potential interference in Full Tau by exon 8 with the microtubule-binding domain, as suggested by the AlphaFold model (**Fig. 4**).

Finally, we analyzed the cell viability and growth rate of Hek-293T cells following infection with lentivirus expressing Tau 42 and Full Tau. Overexpression of Tau 42 significantly decreased the proportion of live cells compared to Full Tau overexpression. These changes suggest that Tau 42, due to its excessive microtubule-stabilizing capacity, more drastically affects cell morphology, adhesion and proliferation than Full Tau, interfering with the normal dynamics necessary for cell division and transport.

A characteristic of mature neurons is the lack of proliferation due to cell cycle exit [49, 50]. Neuronal microtubules are more stable than those in dividing cells [51] and are primarily used for forming complex structures related to their morphology [50]. Stable microtubules in neurons result from the binding of microtubule-associated proteins (MAPs), such as MAP1B, MAP2, or Tau, to brain tubulin, which could facilitate cell cycle exit and reduced cell proliferation. The action of any of these MAPs could complement the absence of other ones. Indeed, mice lacking tau protein are viable although they have some defects [14].

Thus, to analyze the role of the Full Tau in the absence of other brain MAPs, or tau isoforms, we have expressed Full Tau in non-neuronal cells, since in previous studies it was reported that other tau isoforms expressed in non-neuronal cells facilitates microtubule stabilization and reduce cell proliferation [38]. Curiously, Full Tau show different effects as indicated in this work.

In summary, the Full Tau isoform, which contains all exons, shows less interaction capacity with microtubules and lower self-aggregation compared to the Tau 42 isoform. The structural characteristics of Full Tau affect the normal functionality of the Tau protein, suggesting that Full Tau could be detrimental to neuronal stability and viability.

Experimental procedures

Bacteria culture and Tau purification

Vectors pRK-T42 and pRK-FullTau and pRK-T42+E8 (GenScript, Netherlands), encoding different Tau isoforms were overexpressed in *E. coli* BL21 competent cells by electroporation, from which Tau was purified, as described elsewhere [48]. These bacteria were cultured in LB medium with 100 ng/ml of Ampicillin at 37 °C overnight.

Bacterial suspensions were transferred to 1L of LB with 100 ng/ml of Ampicillin and further incubated at 37 °C, until the optical density readings at 600 nm ranged between 0.6 and 0.8. At this point, 0.5 mM of IPTG was added to each sample so as to trigger transcription and incubated once again at 30 °C for 3 h. The samples were centrifuged at 2950×g at 4 °C for 20 min and pellets were resuspended in Tau buffer, which consists of Lysis buffer (20 mM MES pH 6.4, 1mM EGTA, 2 mM DTT) complemented with protease inhibitor mixture, 0.2mM MgCl2, lysozyme and DNAse I. Subsequently, cells were disrupted with a French pressure cell press (in ice cold conditions to avoid protein degradation). In the next step, NaCl was added to a final concentration of 500mM and lysates were boiled for 20 min. Denatured proteins were removed by ultracentrifugation with 127.000 g at 4 °C for 30 min. To this supernatant, 2 mg/mL of streptomycin sulfate was added at 4°C for 15 minutes. The supernatants were kept to get rid of residual DNA. Ammonium sulfate (50-60%) was added to the samples, and they were incubated agitating at 4 °C for at least 1 h. Subsequently, the tau pellet was obtained by centrifugation at 15,000 g at 4°C for 30 minutes. The pellet was resuspended with buffer A (20mM MES pH 6.8, 1 mM EDTA, 2mM DTT, 0.1mM PMSF, 50mM NaCl) and dialyzed overnight at 4 °C against dialysis buffer A to remove salt. Next day, it was treated with RNAse (10mg/mL). Pellets containing purified tau were resuspended in buffer A and the purification process was checked by SDS-PAGE and Coomassie blue staining to check for the whole protein bands appeared upon Coomassie blue staining. The presence of human Tau isoforms was confirmed by Western blot using HT7 antibody (**Supp. Fig. 2**)

Western blots

The proteins were separated on 10% acrylamide/bisacrylamide gels in the presence of SDS at 120 mV for approximately 1 h. The proteins in the gel were transferred to nitrocellulose membranes (Schleicher and Schuell, Keene, NH, USA) at 150 mA for 45 min, using the Bio-Rad Mini-Protean system. Subsequently, the membranes were

blocked using 5% milk powder in 0.1% Tween PBS for 1 h. The membranes were then washed twice with 0.1% PBS Tween-20 (v/v) under stirring for 10 min. The membrane was stained with Ponceau dye (Ponceau 0.3% in TCA 3%) to check the transfer efficiency. Finally, they were incubated with the appropriate primary antibody overnight at 4 °C: anti-human Tau (HT7, mouse, 1:5000, Thermo Scientific); anti-BetaTubulin (mouse, 1:5000, Sigma). Protein expression was detected using the secondary antibody (1:2000), which were incubated per one hour at room temperature. After performing three 10-min washes in the wash solution, the immunoreactive proteins were detected using the ECL (Enhanced Chemiluminescence Detection System, Amersham).

3D modeling analysis

A user-friendly interface for accessing AlphaFold2 has recently been made available through notebooks. We used the ColabFold notebook, whose structure prediction is powered by AlphaFold2 combined with RoseTTAFold and a fast, multiple sequence alignment generation stage using MMseqs2. Each isoform structure prediction was downloaded from ColabFold and visualized with The PyMOL Molecular Graphics System, Version 2.0 Schrödinger, LLC. For accurate visualization, the microtubule binding domain region of both Tau isoforms was colored in red, while the rest of the protein was colored in white. Four images with different rotations along the y-axis were taken to provide a complete visualization of the protein structure.

Tau microtubule-binding capacity assay

Tau microtubule polymerization was carried out as previously described [44, 52], using the brain of 10-month-old C57BL/6 mice. After a first polymerization cycle, the microtubule fraction was collected by centrifugation. The microtubule pellet was resuspended in a buffer containing 0.1M MES, and a second polymerization cycle in the

absence or presence of Full human Tau isoform or human Tau 42 isoform. After incubation for 30 min at 37 °C, electron microscopy [53], and the samples were centrifuged and the protein present in super and pellet fraction were characterized by gel electrophoresis and Western Blot using Tau antibody HT7 and quantified.

Aggregation assays

Tau aggregates were grown for the different Tau isoforms by vapor diffusion in hanging drops, following the standard procedure employed for protein crystallization [54], see also [55] . The different Tau isoforms were purified from bacteria as outlined previously. Their concentration was estimated by measuring absorbance at 280 nm, factoring in individually determined extinction coefficients for each isoform, and confirmed via Coomassie Blue staining.

40 µM of Full Tau or Tau 42 isoforms in a buffer containing 0.1 M NaCl, 20 mM ammonium acetate and 1.5 mM CaCl2, pH 7.0 were incubated in the absence or presence (10 µM) of heparin for 12 h at 37 °C [55]. Protein aggregates were analyzed by electron microscopy [48] and isolated by centrifugation [35], measuring the amount of protein in super and pellet by gel electrophoresis and Western Blot, using Tau antibody HT7, being quantified the obtained results.

Cell Culture

Hek-293T cells (CRL-11268, ATCC) were cultured in DMEM supplemented with 10% fetal bovine serum, 2 mM glutamine, non-essential amino acids, 10 U/ml penicillin and 10 µg/ml streptomycin, at 37 °C and 5% CO_2.

Lentivirus Production

Viral stocks were produced in P100 plates by transient cotransfection of Hek-293T cells with 10 µg of the corresponding lentivector plasmid: Lv Tau 42 (kindly provided by Kenneth Kosik, UC Santa Barbara, California), Lv- Empty Tau 42 (GFP; Addgene plasmid 12252), Lv- Full Tau (GenScript, Netherlands) and Lv- Empty Full Tau (qWPI; Addgene plasmid #12254), 6 µg of the packaging plasmid pCMVdR8.74 (Addgene plasmid 22036) and 5 µg of the VSV-G envelope protein plasmid pMD2G (Addgene plasmid 12259) using Lipofectamine LTX reagent (Invitrogen) and PLUS REagents following instructions of the supplier (Invitrogen). Next day, the transfection was stopped and 5 mL of medium were added. Two and three days after transfection, lentivirus were collected and stored at -80 ºC. Lentiviral titer was calculated as previously described [56].

Study of cell viability and growth rate in non-neuronal cells expressing human tau isoforms

HEK-203T cells were infected by packaged lentiviral particles at an approximated MOI of 5 Infective Units (IU)/cell. Forty-eight and seventy-two hours after infection, photos were taken with the Orca Flash (monochrome sCMOS) (Zeiss) using a 40X dry objective under brightfield illumination. Western blot was performed for infection confirmation. Viability assay was performed following the Calcein AM assay. 2 μM calcein AM working solution in PBS was added to the cells. The plates were incubated at 37 °C for 20 minutes, and calcein AM fluorescence (ex/em 494/517 nm) was read by sCMOS ORCA-Fusion (C11440-20UP) using a 10X/0.3 Aire EC Plan-Neo (Zeiss 420340-9901). The quantification of growth rate and viability was performed using Fiji [57]. Quantitative analysis was done using One-way ANOVA followed by Student's t-test for comparisons.

Statistical analysis

For quantitative experiments, the statistical significance was determined by Student’s t test or One-way ANOVA with Turkey post hoc test, for comparisons including more than two groups. A p value of less than 0.05 was considered significant (* $p < 0.05$; ** $p < 0.01$). Data analysis was conducted using GraphPad Prism (GraphPad Software, La Jolla, CA, USA). For all figures in which error bars are shown, data represent the mean ± SEM. The normality of the data distribution was assessed using the Shapiro-Wilk test.

Supporting information

This article contains supporting information

Acknowledgments

Thanks to the SMOA and Electron Microscopy services of the CBMSO for the resources, knowledge and time dedicated to the achievement of this project. We thank Ms. Nuria de la Torre Alonso for technical and editorial assistance

Author contributions

Conceptualization: LVS, IDS, FH and JA;
Data curation: LVS and IDS;
Formal analysis: LVS, IDS, FH and JA;
Funding acquisition: FH and JA;
Investigation: LVS and IDS;
Methodology: LVS, IDS, AJP, MP and VGE;

Project Writing – original draft: LVS, IDS, FH and JA;

Writing – review and editing: LVS, IDS, AJP, MP, VGE, FH and JA

Data Availability

Data is available in data repository: https://doi.org/10.6084/m9.figshare.28351508.v1

Funding and additional information

This work has been supported by grants from the Spanish Ministry of Science: PID2020-113204GB-I00 (F.H.) and PID2021-123859OB-100 from MCIN /AEI/10.13039/501100011033 / FEDER, UE (J.A.). The Centro de Biología Molecular Severo Ochoa (CBMSO) is a Severo Ochoa Center of Excellence (MICIN, award CEX2021-001154-S).

Conflict of interest

The authors declare that they have no conflicts of interest with the contents of this article.

References

1. Goedert M, Crowther RA, Garner CC. Molecular characterization of microtubule-associated proteins tau and MAP2. Trends Neurosci. 1991;14(5):193-9. doi: 10.1016/0166-2236(91)90105-4. PubMed PMID: 1713721.
2. Weingarten MD, Lockwood AH, Hwo SY, Kirschner MW. A protein factor essential for microtubule assembly. Proc Natl Acad Sci U S A. 1975;72(5):1858-62. doi: 10.1073/pnas.72.5.1858. PubMed PMID: 1057175; PubMed Central PMCID: PMCPMC432646.

3. Baas PW, Qiang L. Tau: It's Not What You Think. Trends Cell Biol. 2019;29(6):452-61. Epub 20190328. doi: 10.1016/j.tcb.2019.02.007. PubMed PMID: 30929793; PubMed Central PMCID: PMCPMC6527491.

4. Avila J, Lucas JJ, Perez M, Hernandez F. Role of tau protein in both physiological and pathological conditions. Physiol Rev. 2004;84(2):361-84. doi: 10.1152/physrev.00024.2003. PubMed PMID: 15044677.

5. Corsi A, Bombieri C, Valenti MT, Romanelli MG. Tau Isoforms: Gaining Insight into MAPT Alternative Splicing. Int J Mol Sci. 2022;23(23). Epub 20221206. doi: 10.3390/ijms232315383. PubMed PMID: 36499709; PubMed Central PMCID: PMCPMC9735940.

6. Fischer I, Baas PW. Resurrecting the Mysteries of Big Tau. Trends Neurosci. 2020;43(7):493-504. Epub 20200517. doi: 10.1016/j.tins.2020.04.007. PubMed PMID: 32434664; PubMed Central PMCID: PMCPMC7999525.

7. Goedert M, Jakes R, Spillantini MG, Hasegawa M, Smith MJ, Crowther RA. Assembly of microtubule-associated protein tau into Alzheimer-like filaments induced by sulphated glycosaminoglycans. Nature. 1996;383(6600):550-3. doi: 10.1038/383550a0. PubMed PMID: 8849730.

8. Guo T, Noble W, Hanger DP. Roles of tau protein in health and disease. Acta Neuropathologica. 2017;133(5):665-704. doi: 10.1007/s00401-017-1707-9.

9. Iqbal K, Liu F, Gong CX. Tau and neurodegenerative disease: the story so far. Nat Rev Neurol. 2016;12(1):15-27. Epub 20151204. doi: 10.1038/nrneurol.2015.225. PubMed PMID: 26635213.

10. Neve RL, Harris P, Kosik KS, Kurnit DM, Donlon TA. Identification of cDNA clones for the human microtubule-associated protein tau and chromosomal localization of the genes for tau and microtubule-associated protein 2. Brain Res. 1986;387(3):271-80. doi: 10.1016/0169-328x(86)90033-1. PubMed PMID: 3103857.

11. Andreadis A. Tau gene alternative splicing: expression patterns, regulation and modulation of function in normal brain and neurodegenerative diseases. Biochim

Biophys Acta. 2005;1739(2-3):91-103. doi: 10.1016/j.bbadis.2004.08.010. PubMed PMID: 15615629.

12. Caillet-Boudin ML, Buee L, Sergeant N, Lefebvre B. Regulation of human MAPT gene expression. Mol Neurodegener. 2015;10:28. Epub 20150714. doi: 10.1186/s13024-015-0025-8. PubMed PMID: 26170022; PubMed Central PMCID: PMCPMC4499907.

13. Liu C, Gotz J. Profiling murine tau with 0N, 1N and 2N isoform-specific antibodies in brain and peripheral organs reveals distinct subcellular localization, with the 1N isoform being enriched in the nucleus. PLoS One. 2013;8(12):e84849. Epub 20131230. doi: 10.1371/journal.pone.0084849. PubMed PMID: 24386422; PubMed Central PMCID: PMCPMC3875548.

14. Buchholz S, Zempel H. The six brain-specific TAU isoforms and their role in Alzheimer's disease and related neurodegenerative dementia syndromes. Alzheimers Dement. 2024;20(5):3606-28. Epub 20240331. doi: 10.1002/alz.13784. PubMed PMID: 38556838; PubMed Central PMCID: PMCPMC11095451.

15. Tress ML, Abascal F, Valencia A. Alternative Splicing May Not Be the Key to Proteome Complexity. Trends Biochem Sci. 2017;42(2):98-110. Epub 20161003. doi: 10.1016/j.tibs.2016.08.008. PubMed PMID: 27712956; PubMed Central PMCID: PMCPMC6526280.

16. Garcia-Escudero V, Ruiz-Gabarre D, Gargini R, Perez M, Garcia E, Cuadros R, et al. A new non-aggregative splicing isoform of human Tau is decreased in Alzheimer's disease. Acta Neuropathol. 2021;142(1):159-77. Epub 20210502. doi: 10.1007/s00401-021-02317-z. PubMed PMID: 33934221; PubMed Central PMCID: PMCPMC8217066.

17. Ngian ZK, Tan YY, Choo CT, Lin WQ, Leow CY, Mah SJ, et al. Truncated Tau caused by intron retention is enriched in Alzheimer's disease cortex and exhibits altered biochemical properties. Proc Natl Acad Sci U S A. 2022;119(37):e2204179119. Epub 20220906. doi: 10.1073/pnas.2204179119. PubMed PMID: 36067305; PubMed Central PMCID: PMCPMC9477417.

18. Ruiz-Gabarre D, Valles-Saiz L, Carnero-Espejo A, Ferrer I, Hernandez F, Garcia-Escudero R, et al. Intron retention as a productive mechanism in human MAPT: RNA species generated by retention of intron 3. EBioMedicine. 2024;100:104953. Epub 20240105. doi: 10.1016/j.ebiom.2023.104953. PubMed PMID: 38181704; PubMed Central PMCID: PMCPMC10789595.

19. Butler SJ, Bronner ME. From classical to current: analyzing peripheral nervous system and spinal cord lineage and fate. Dev Biol. 2015;398(2):135-46. Epub 20141024. doi: 10.1016/j.ydbio.2014.09.033. PubMed PMID: 25446276; PubMed Central PMCID: PMCPMC4845735.

20. Ridet JL, Perre J, Foncin JF. [The transition zone of the central nervous system-peripheral nervous system of the adult rat; ultrastructural and immunocytochemical studies: a new function of the astroglia?]. J Hirnforsch. 1991;32(6):761-4. PubMed PMID: 1726542.

21. Nothias F, Boyne L, Murray M, Tessler A, Fischer I. The expression and distribution of tau proteins and messenger RNA in rat dorsal root ganglion neurons during development and regeneration. Neuroscience. 1995;66(3):707-19. doi: 10.1016/0306-4522(94)00598-y. PubMed PMID: 7644032.

22. Couchie D, Mavilia C, Georgieff IS, Liem RK, Shelanski ML, Nunez J. Primary structure of high molecular weight tau present in the peripheral nervous system. Proc Natl Acad Sci U S A. 1992;89(10):4378-81. doi: 10.1073/pnas.89.10.4378. PubMed PMID: 1374898; PubMed Central PMCID: PMCPMC49085.

23. Goedert M, Spillantini MG, Crowther RA. Cloning of a big tau microtubule-associated protein characteristic of the peripheral nervous system. Proc Natl Acad Sci U S A. 1992;89(5):1983-7. doi: 10.1073/pnas.89.5.1983. PubMed PMID: 1542696; PubMed Central PMCID: PMCPMC48578.

24. Chen WT, Liu WK, Yen SH. Expression of tau exon 8 in different species. Neurosci Lett. 1994;172(1-2):167-70. doi: 10.1016/0304-3940(94)90688-2. PubMed PMID: 8084527.

25. Himmler A. Structure of the bovine tau gene: alternatively spliced transcripts generate a protein family. Mol Cell Biol. 1989;9(4):1389-96. doi: 10.1128/mcb.9.4.1389-1396.1989. PubMed PMID: 2498650; PubMed Central PMCID: PMCPMC362555.

26. Andreadis A, Nisson PE, Kosik KS, Watkins PC. The exon trapping assay partly discriminates against alternatively spliced exons. Nucleic Acids Res. 1993;21(9):2217-21. doi: 10.1093/nar/21.9.2217. PubMed PMID: 7684835; PubMed Central PMCID: PMCPMC309487.

27. Nelson PT, Stefansson K, Gulcher J, Saper CB. Molecular evolution of tau protein: implications for Alzheimer's disease. J Neurochem. 1996;67(4):1622-32. doi: 10.1046/j.1471-4159.1996.67041622.x. PubMed PMID: 8858947.

28. Gendron TF, Petrucelli L. The role of tau in neurodegeneration. Mol Neurodegener. 2009;4:13. Epub 20090311. doi: 10.1186/1750-1326-4-13. PubMed PMID: 19284597; PubMed Central PMCID: PMCPMC2663562.

29. Chakraborty P, Riviere G, Liu S, de Opakua AI, Dervisoglu R, Hebestreit A, et al. Co-factor-free aggregation of tau into seeding-competent RNA-sequestering amyloid fibrils. Nat Commun. 2021;12(1):4231. Epub 20210709. doi: 10.1038/s41467-021-24362-8. PubMed PMID: 34244499; PubMed Central PMCID: PMCPMC8270918.

30. Cleveland DW, Hwo SY, Kirschner MW. Purification of tau, a microtubule-associated protein that induces assembly of microtubules from purified tubulin. J Mol Biol. 1977;116(2):207-25. doi: 10.1016/0022-2836(77)90213-3. PubMed PMID: 599557.

31. Makrides V, Massie MR, Feinstein SC, Lew J. Evidence for two distinct binding sites for tau on microtubules. Proc Natl Acad Sci U S A. 2004;101(17):6746-51. Epub 20040419. doi: 10.1073/pnas.0400992101. PubMed PMID: 15096589; PubMed Central PMCID: PMCPMC404116.

32. Fischer I. Big Tau: What We Know, and We Need to Know. eNeuro. 2023;10(5). Epub 20230510. doi: 10.1523/ENEURO.0052-23.2023. PubMed PMID: 37164636; PubMed Central PMCID: PMCPMC10179842.

33. Bourdenx M, Koulakiotis NS, Sanoudou D, Bezard E, Dehay B, Tsarbopoulos A. Protein aggregation and neurodegeneration in prototypical neurodegenerative diseases: Examples of amyloidopathies, tauopathies and synucleinopathies. Prog Neurobiol. 2017;155:171-93. Epub 20150721. doi: 10.1016/j.pneurobio.2015.07.003. PubMed PMID: 26209472.

34. Lee VM, Goedert M, Trojanowski JQ. Neurodegenerative tauopathies. Annu Rev Neurosci. 2001;24:1121-59. doi: 10.1146/annurev.neuro.24.1.1121. PubMed PMID: 11520930.

35. Perez M, Valpuesta JM, Medina M, Montejo de Garcini E, Avila J. Polymerization of tau into filaments in the presence of heparin: the minimal sequence required for tau-tau interaction. J Neurochem. 1996;67(3):1183-90. doi: 10.1046/j.1471-4159.1996.67031183.x. PubMed PMID: 8752125.

36. Jumper J, Evans R, Pritzel A, Green T, Figurnov M, Ronneberger O, et al. Highly accurate protein structure prediction with AlphaFold. Nature. 2021;596(7873):583-9. Epub 20210715. doi: 10.1038/s41586-021-03819-2. PubMed PMID: 34265844; PubMed Central PMCID: PMCPMC8371605.

37. Avila J. Microtubule functions. Life Sci. 1992;50(5):327-34. doi: 10.1016/0024-3205(92)90433-p. PubMed PMID: 1732704.

38. Lee G, Rook SL. Expression of tau protein in non-neuronal cells: microtubule binding and stabilization. J Cell Sci. 1992;102 (Pt 2):227-37. doi: 10.1242/jcs.102.2.227. PubMed PMID: 1400630.

39. Sanchez C, Diaz-Nido J, Avila J. Phosphorylation of microtubule-associated protein 2 (MAP2) and its relevance for the regulation of the neuronal cytoskeleton function. Prog Neurobiol. 2000;61(2):133-68. doi: 10.1016/s0301-0082(99)00046-5. PubMed PMID: 10704996.

40. Tucker RP. The roles of microtubule-associated proteins in brain morphogenesis: a review. Brain Res Brain Res Rev. 1990;15(2):101-20. doi: 10.1016/0165-0173(90)90013-e. PubMed PMID: 2282447.

41. Matus A. Microtubule-associated proteins: their potential role in determining neuronal morphology. Annu Rev Neurosci. 1988;11:29-44. doi: 10.1146/annurev.ne.11.030188.000333. PubMed PMID: 3284444.

42. Barbier P, Zejneli O, Martinho M, Lasorsa A, Belle V, Smet-Nocca C, et al. Role of Tau as a Microtubule-Associated Protein: Structural and Functional Aspects. Front Aging Neurosci. 2019;11:204. Epub 20190807. doi: 10.3389/fnagi.2019.00204. PubMed PMID: 31447664; PubMed Central PMCID: PMCPMC6692637.

43. El Mammeri N, Dregni AJ, Duan P, Wang HK, Hong M. Microtubule-binding core of the tau protein. Sci Adv. 2022;8(29):eabo4459. Epub 20220720. doi: 10.1126/sciadv.abo4459. PubMed PMID: 35857846; PubMed Central PMCID: PMCPMC9299549.

44. Serrano L, de la Torre J, Maccioni RB, Avila J. Involvement of the carboxyl-terminal domain of tubulin in the regulation of its assembly. Proc Natl Acad Sci U S A. 1984;81(19):5989-93. doi: 10.1073/pnas.81.19.5989. PubMed PMID: 6385005; PubMed Central PMCID: PMCPMC391844.

45. Serrano L, Montejo de Garcini E, Hernandez MA, Avila J. Localization of the tubulin binding site for tau protein. Eur J Biochem. 1985;153(3):595-600. doi: 10.1111/j.1432-1033.1985.tb09342.x. PubMed PMID: 3935441.

46. Kellogg EH, Hejab NMA, Poepsel S, Downing KH, DiMaio F, Nogales E. Near-atomic model of microtubule-tau interactions. Science. 2018;360(6394):1242-6. Epub 20180510. doi: 10.1126/science.aat1780. PubMed PMID: 29748322; PubMed Central PMCID: PMCPMC6225777.

47. Stancu IC, Ferraiolo M, Terwel D, Dewachter I. Tau Interacting Proteins: Gaining Insight into the Roles of Tau in Health and Disease. Adv Exp Med Biol. 2019;1184:145-66. doi: 10.1007/978-981-32-9358-8_13. PubMed PMID: 32096036.

48. Perez M, Arrasate M, Montejo De Garcini E, Munoz V, Avila J. In vitro assembly of tau protein: mapping the regions involved in filament formation. Biochemistry. 2001;40(20):5983-91. doi: 10.1021/bi002961w. PubMed PMID: 11352733.

49. Homem CC, Repic M, Knoblich JA. Proliferation control in neural stem and progenitor cells. Nat Rev Neurosci. 2015;16(11):647-59. Epub 20150930. doi: 10.1038/nrn4021. PubMed PMID: 26420377; PubMed Central PMCID: PMCPMC4667397.

50. Sakakibara A, Ando R, Sapir T, Tanaka T. Microtubule dynamics in neuronal morphogenesis. Open Biol. 2013;3(7):130061. Epub 20130717. doi: 10.1098/rsob.130061. PubMed PMID: 23864552; PubMed Central PMCID: PMCPMC3728923.

51. Baas PW, Rao AN, Matamoros AJ, Leo L. Stability properties of neuronal microtubules. Cytoskeleton (Hoboken). 2016;73(9):442-60. doi: 10.1002/cm.21286. PubMed PMID: 26887570; PubMed Central PMCID: PMCPMC5541393.

52. Serrano L, Valencia A, Caballero R, Avila J. Localization of the high affinity calcium-binding site on tubulin molecule. J Biol Chem. 1986;261(15):7076-81. PubMed PMID: 3700428.

53. Gaskin F, Cantor CR, Shelanski ML. Turbidimetric studies of the in vitro assembly and disassembly of porcine neurotubules. J Mol Biol. 1974;89(4):737-55. doi: 10.1016/0022-2836(74)90048-5. PubMed PMID: 4475698.

54. Crowther RA, Olesen OF, Smith MJ, Jakes R, Goedert M. Assembly of Alzheimer-like filaments from full-length tau protein. FEBS Lett. 1994;337(2):135-8. doi: 10.1016/0014-5793(94)80260-2. PubMed PMID: 8287967.

55. Fichou Y, Lin Y, Rauch JN, Vigers M, Zeng Z, Srivastava M, et al. Cofactors are essential constituents of stable and seeding-active tau fibrils. Proc Natl Acad Sci U S A. 2018;115(52):13234-9. Epub 20181211. doi: 10.1073/pnas.1810058115. PubMed PMID: 30538196; PubMed Central PMCID: PMCPMC6310788.

56. Martin-Maestro P, Gargini R, Garcia E, Simon D, Avila J, Garcia-Escudero V. Mitophagy Failure in APP and Tau Overexpression Model of Alzheimer's Disease. J Alzheimers Dis. 2019;70(2):525-40. doi: 10.3233/JAD-190086. PubMed PMID: 31256128.

57. Schindelin J, Arganda-Carreras I, Frise E, Kaynig V, Longair M, Pietzsch T, et al. Fiji: an open-source platform for biological-image analysis. Nat Methods. 2012;9(7):676-82. Epub 20120628. doi: 10.1038/nmeth.2019. PubMed PMID: 22743772; PubMed Central PMCID: PMCPMC3855844.

Abbreviations

AD: Alzheimer's disease

AF2: AlphaFold 2

CNS: central nervous system

MAPs: microtubule-associated proteins

MTs: Mouse brain microtubules

PNS: Peripheral Nervous System

Figure 1 Click here to access/download;Figure;Fig1.tif

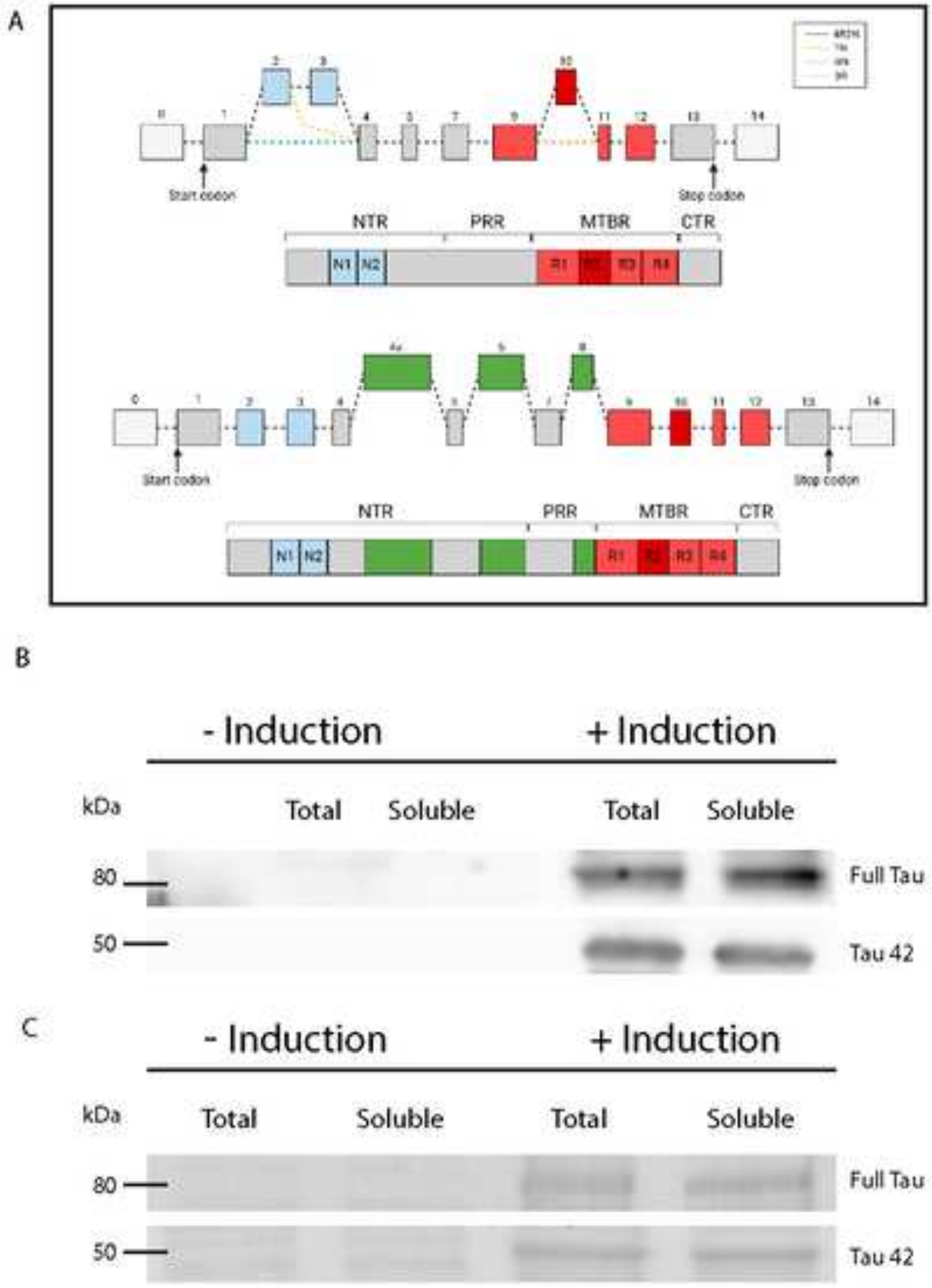

Figure 2

Click here to access/download;Figure;Fig2.tif

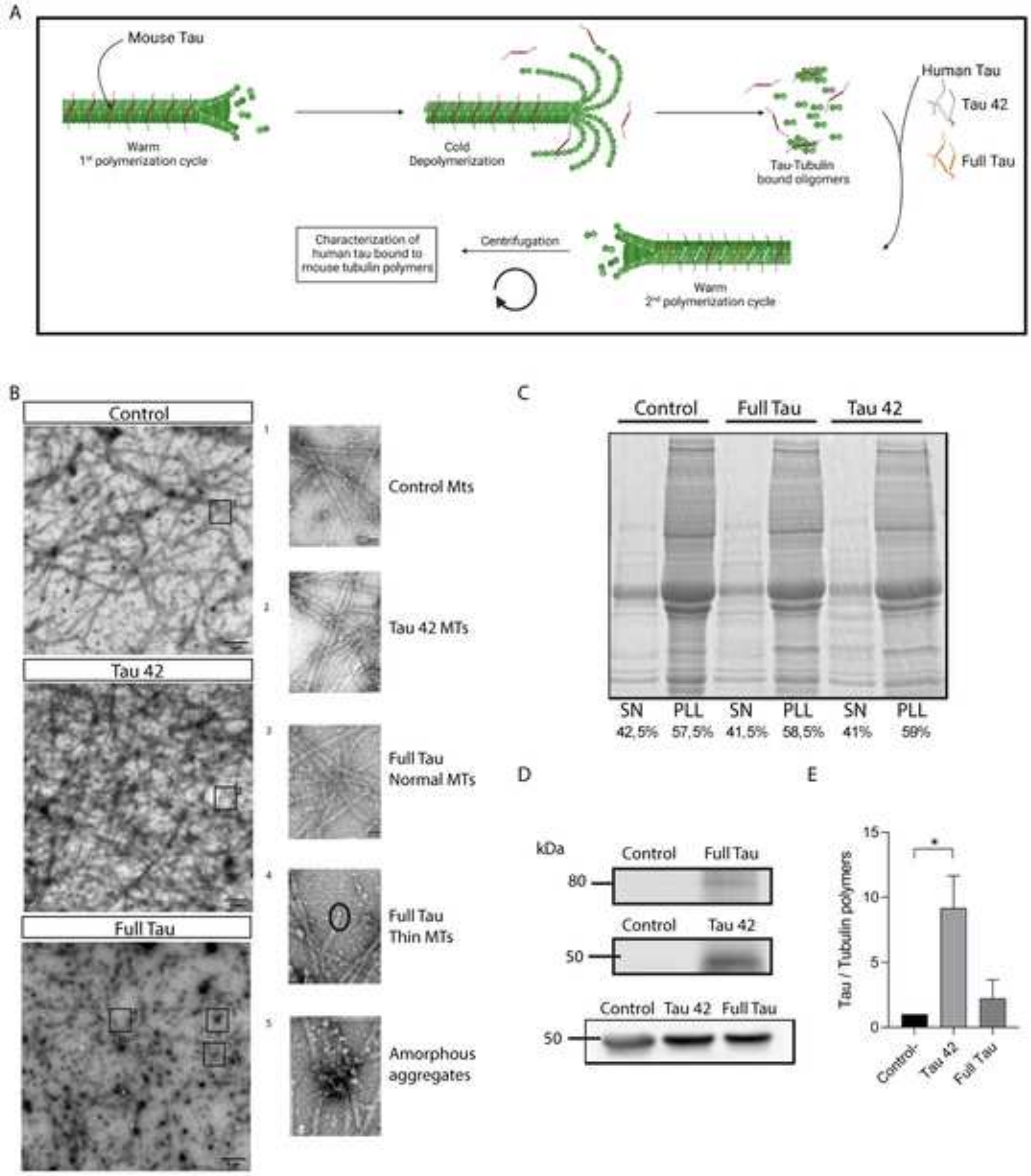

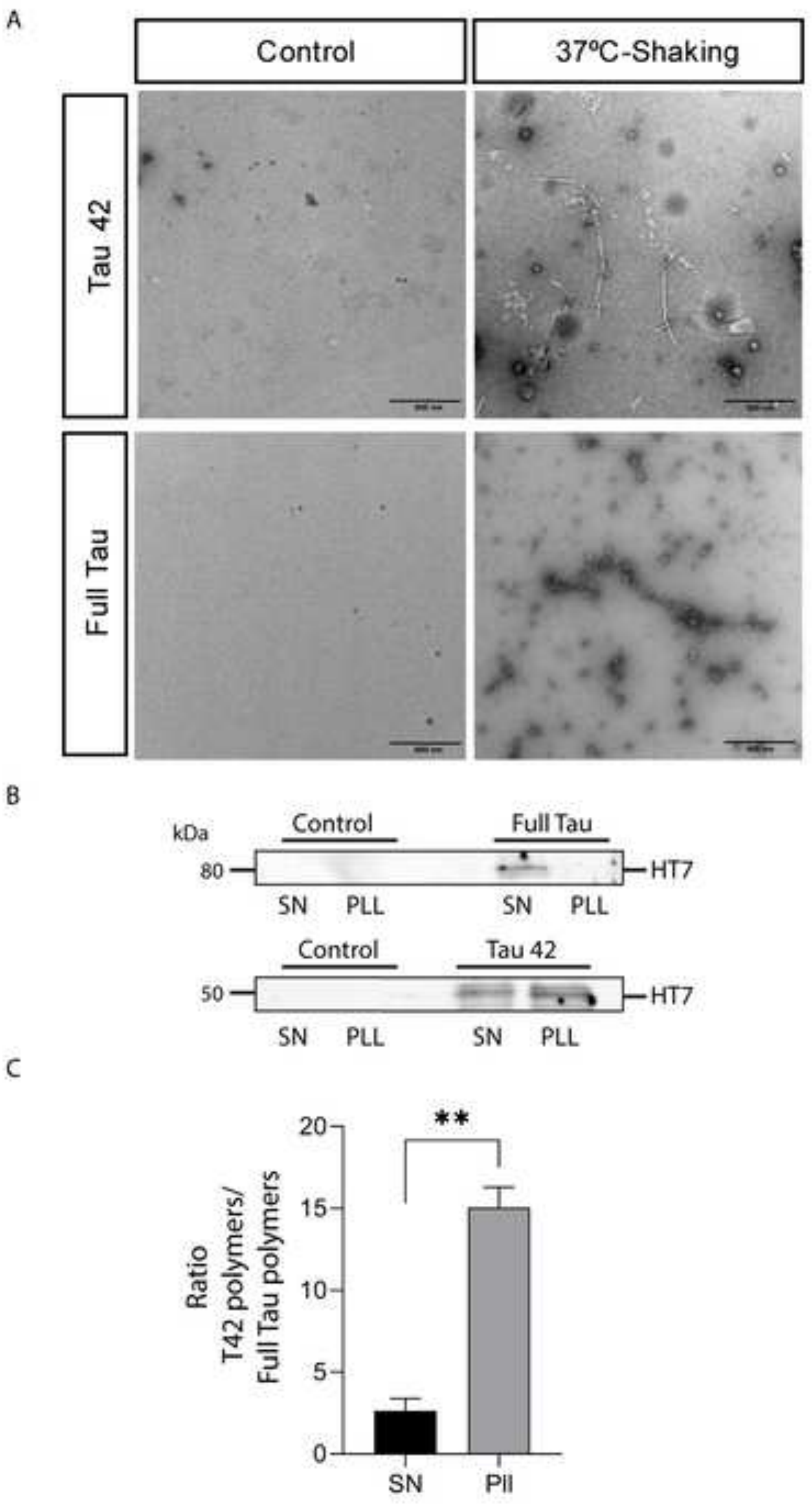
A
Control
37ºC-Shaking
Tau 42
Full Tau
B
kDa
Control
Full Tau
80
HT7
SN
PLL
SN
PLL
Control
Tau 42
50
HT7
SN
PLL
SN
PLL
C
Ratio T42 polymers/ Full Tau polymers
20
15
10
5
0
**
SN
Pll

Figure 4

Click here to access/download;Figure;Fig4.tif

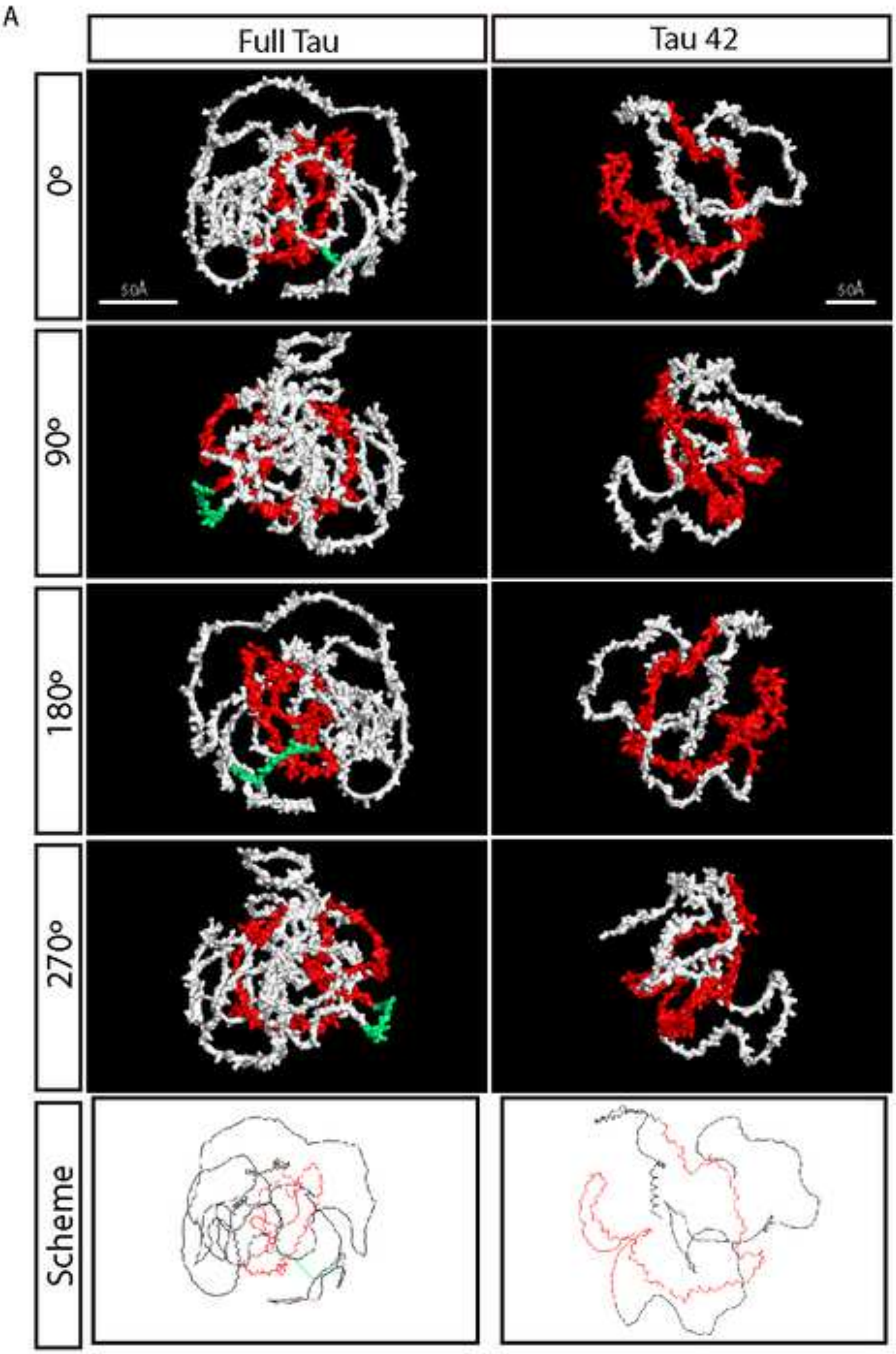

Figure 5

Click here to access/download;Figure;Fig5.tif

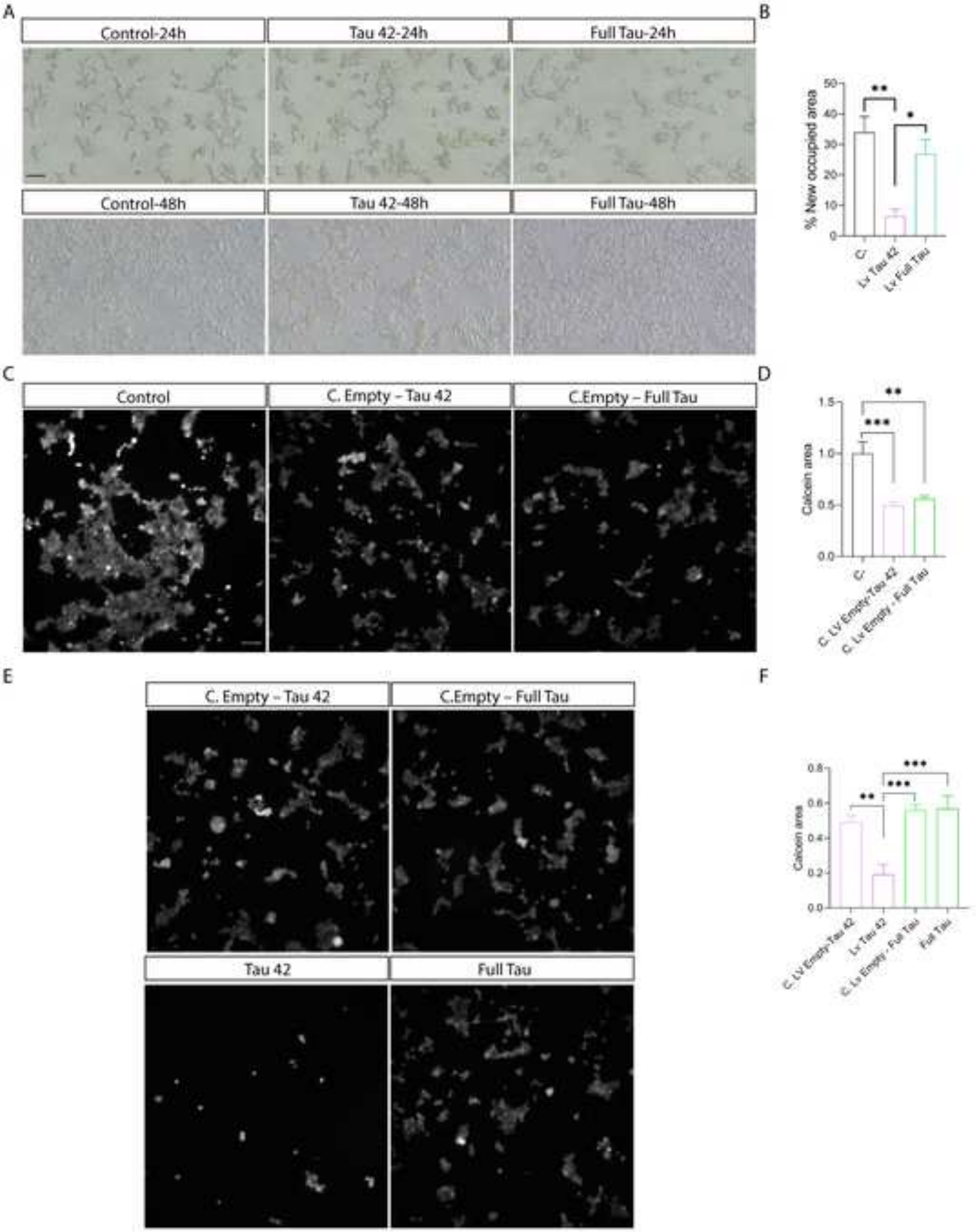

Click here to access/download
Supporting Information
SupplementaryMaterial.pdf
