# Supplementary material for: Structure and function of Full-length Tau
Source: PLoS One. 2025 Oct 31;20(10):e0335251. doi: 10.1371/journal.pone.0335251 (PMC12578172; doi:10.1371/journal.pone.0335251)
Supplement: Attachment — Submitted filename: Response to Editors.docx [file pone.0335251.s012.docx]

Dear Prof. Ginsberg,

Editor Plos One.

Please you will find enclosed the revised manuscript entitled “Structure and function of Full-length Tau.”

In this version, we have included unadjusted and uncropped images of blots and gels that are found on Supporting Information and publicly available data repository figshare, as S1_raw_images.pdf:

Vallés-Saiz, Laura; Domene Serrano, Indalo; Picher, Angel J; Pérez, Mar; García-Escudero, Vega; Felix, Hernandez; et al. (2025). Structure and Function of full-lenght Tau. figshare. Dataset. <https://doi.org/10.6084/m9.figshare.28351508.v5>.

We hope we have adequately addressed your requests. If not, please specify precisely which changes you would like to see.

Yours sincerely,
